# Noncanonical prokaryotic X family DNA polymerases lack polymerase activity and act as exonucleases

**DOI:** 10.1093/nar/gkac461

**Published:** 2022-06-03

**Authors:** Maria Prostova, Evgeniy Shilkin, Alexandra A Kulikova, Alena Makarova, Sergei Ryazansky, Andrey Kulbachinskiy

**Affiliations:** Institute of Molecular Genetics, National Research Centre “Kurchatov Institute”, Moscow 123182, Russia; Institute of Molecular Genetics, National Research Centre “Kurchatov Institute”, Moscow 123182, Russia; Institute of Molecular Genetics, National Research Centre “Kurchatov Institute”, Moscow 123182, Russia; Institute of Molecular Genetics, National Research Centre “Kurchatov Institute”, Moscow 123182, Russia; Institute of Molecular Genetics, National Research Centre “Kurchatov Institute”, Moscow 123182, Russia; Institute of Molecular Genetics, National Research Centre “Kurchatov Institute”, Moscow 123182, Russia

## Abstract

The X family polymerases (PolXs) are specialized DNA polymerases that are found in all domains of life. While the main representatives of eukaryotic PolXs, which have dedicated functions in DNA repair, were studied in much detail, the functions and diversity of prokaryotic PolXs have remained largely unexplored. Here, by combining a comprehensive bioinformatic analysis of prokaryotic PolXs and biochemical experiments involving selected recombinant enzymes, we reveal a previously unrecognized group of PolXs that seem to be lacking DNA polymerase activity. The noncanonical PolXs contain substitutions of the key catalytic residues and deletions in their polymerase and dNTP binding sites in the palm and fingers domains, but contain functional nuclease domains, similar to canonical PolXs. We demonstrate that representative noncanonical PolXs from the *Deinococcus* genus are indeed inactive as DNA polymerases but are highly efficient as 3′-5′ exonucleases. We show that both canonical and noncanonical PolXs are often encoded together with the components of the non-homologous end joining pathway and may therefore participate in double-strand break repair, suggesting an evolutionary conservation of this PolX function. This is a remarkable example of polymerases that have lost their main polymerase activity, but retain accessory functions in DNA processing and repair.

## INTRODUCTION

Specialized DNA polymerases of the X family (PolXs) play essential functions in DNA repair and recombination in eukaryotes (1–4). The first eukaryotic PolX polymerases, mammalian polymerase β (Polβ) and terminal deoxynucleotidyltransferase (TdT), were discovered five decades ago (5–13). In 2000s, the family of eukaryotic PolXs was expanded by Polλ and Polμ (14–17) and, more recently, by new PolX variants from plants, marine animals, fungi and viruses (13,18–20). The existence of bacterial and archaeal Polβ-like polymerases was first bioinformatically predicted twenty years ago (21,22). However, to date only four bacterial PolX polymerases from *Bacillus subtilis*, *Thermus thermophilus, Staphylococcus aureus* and *Deinococcus radiodurans* have been partially characterized, and none of the archaea (23–26).

**Figure 1.**
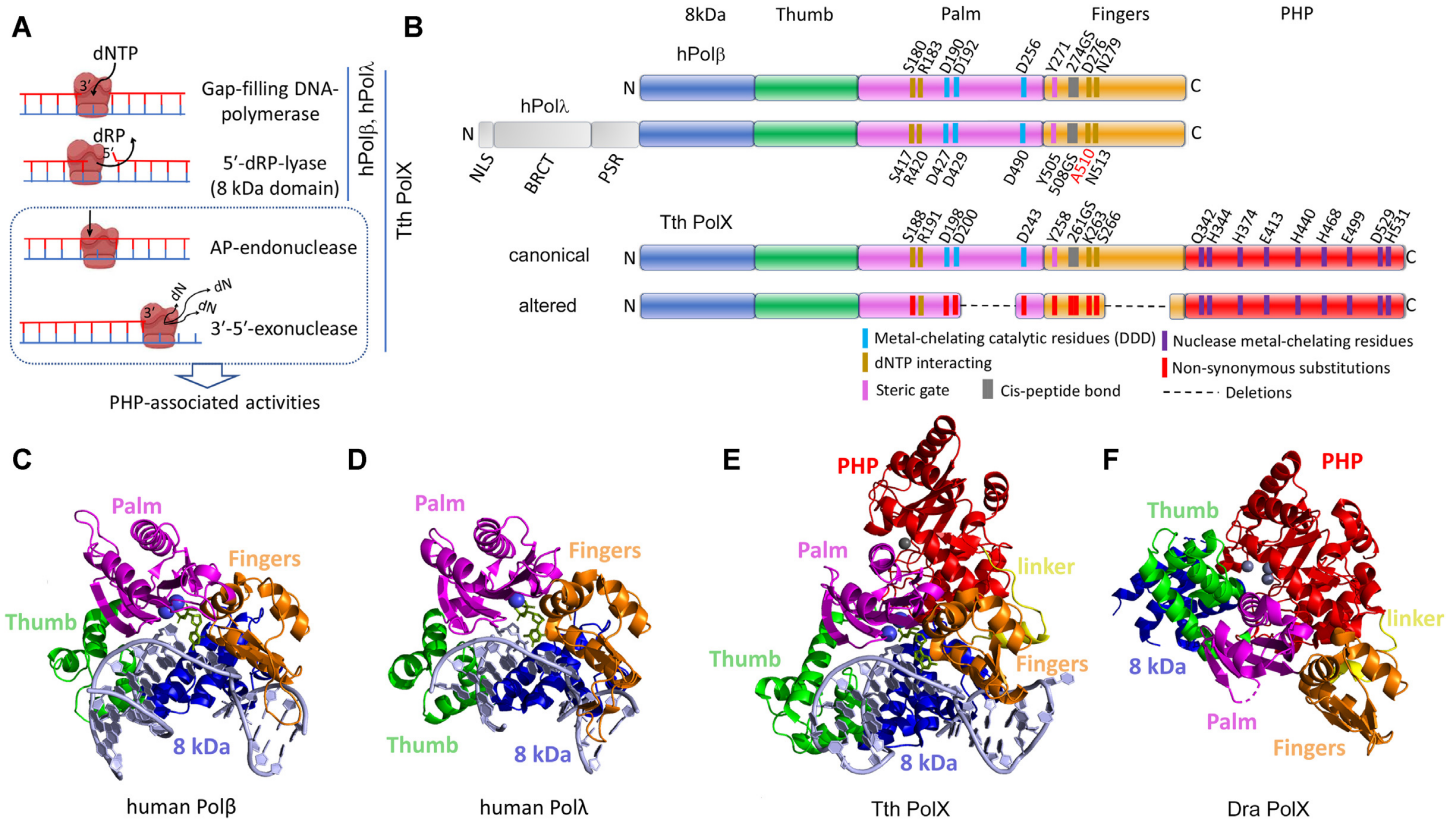
The structure and functions of prokaryotic and human PolXs. (**A**) Activities of human Polβ, Polλ and *T. thermophilus* (Tth) PolX. (**B**) The overall domain structure of human Polβ, Polλ, canonical prokaryotic PolXs (illustrated for Tth PolX) and altered prokaryotic PolXs. The N-terminal lyase domain is shown in blue, the thumb domain is green, the palm domain is pink, the fingers domain is orange, the PHP domain is red. The N-terminal part of Polλ, including the nuclear localization signal (NLS), the BRCA1 C-terminal (BRCT) and proline-serine rich (PSR) domains, is shown in gray. The active site residues of the palm and PHP domains are marked in blue and violet in canonical PolXs (the numbering is for *T. thermophilus* PolX) and in red in altered PolXs. (**C**) Structure of human Polβ in complex with gapped DNA (PDB: 1BPX). (**D**) Structure of the catalytic core of human Polλ in complex with gapped DNA (PDB: 1XSN). (**E**) Structure of*T. thermophilus* PolX in complex with gapped DNA (PDB: 3AU0). (**F**) Structure of *D. radiodurans* (Dra) PolX (PDB: 2W9M). PolXs are roughly aligned by their palm domains. The active site magnesium in the palm domain is shown with blue spheres; metal ions bound in the exonuclease PHP domain are shown in gray.

Eukaryotic PolXs take part in base excision repair (BER) and double-strand break (DSB) repair during non-homologous end joining (NHEJ) and V(D)J recombination. The activities of these polymerases *in vitro*, which are likely important for their *in vivo* functions, include gap-filling DNA polymerase and dRP-lyase activities (found in Polβ, Polλ and Polμ) (Figure 1A) and template-independent polymerase and end-bridging activities (found in TdT, Polλ and Polμ) (3,27–30). Similarly to Polβ, prokaryotic PolXs have the gap-filling activity and likely the 5′-dRP-lyase activity (23,25,31,32). In addition, prokaryotic polymerases were reported to possess a 3′-5′ exonuclease activity that may play a role in DNA replication and repair, and an AP-endonuclease activity potentially involved in the BER pathway (Figure 1A) (23,25,33,34).

The characteristic feature of PolX polymerases is the presence of the conserved Polβ fold in their catalytic palm domain, which belongs to the ancient superfamily of nucleotidyltransferases (a variant of this fold is also found in the C family of DNA polymerases) (21,35,36). Similar to other DNA polymerases, all PolXs form a common “right hand” structure, with the central polymerase part consisting of the thumb, palm and fingers domains involved in DNA and dNTP binding and catalysis (Figure 1B). In both eukaryotic and prokaryotic PolXs, these domains are preceded by an N-terminal ‘8 kDa’ domain, responsible for the 5′-dRP-lyase activity (31,37). Eukaryotic Polλ, Polμ and TdT additionally contain a BRCA1 C-terminal (BRCT) domain, which is involved in interactions with other proteins during NHEJ and V(D)J recombination (Figure 1B) (14,38–40). In contrast, all studied prokaryotic PolXs contain a C-terminal polymerase and histidinol phosphatase (PHP) domain, which is responsible for the 3′-5′ exonuclease and AP-endonuclease activities and is absent in eukaryotic PolXs (Figure 1B and D) (23,25,33,41). During DNA polymerization, the N-terminal, thumb, palm and fingers domains embrace the DNA substrate to bend it and position the template nucleotide into the active site, as seen in the complexes of human Pol β, Pol λ and *T. thermophilus* PolX with gapped DNA substrates (Figure 1C, D and E) (32,42,43).

Despite the large number of sequenced prokaryotic genomes, the diversity of bacterial and archaeal PolXs have remained largely uninvestigated. Biological functions of PolXs in prokaryotes, their potential roles in various DNA repair pathways, and their interactomes remain mostly unknown. In this study, we present an in depth bioinformatic analysis of prokaryotic PolXs and interpret it in the context of structural and biochemical data available for bacterial polymerases of this family. We show that most prokaryotic PolXs share a common domain architecture but a significant part of them have a noncanonical structure of the active site and probably lack the polymerization activity. At the same time, the noncanonical PolXs contain the highly conserved PHP exonuclease domain and the predicted N-terminal lyase domain. Our findings are corroborated by biochemical analysis of two noncanonical polymerases from *Deinococcus* species. We reveal a possible association of PolX genes with components of the non-homologous end joining pathway and propose that prokaryotic PolX polymerases likely have accessory functions in DNA repair beyond DNA synthesis.

## MATERIALS AND METHODS

### Sequence and genomic analyses of prokaryotic PolXs and phylogenetic trees

The set of proteins, genomic sequences and annotations of prokaryotic genomes were fetched from the NCBI FTP site in December 2019. The search for prokaryotic PolX sequences was performed using the sequences of *B. subtilis*, *T. thermophilus* and *D. radiodurans* PolXs as queries. The identification of homologs of known PolX proteins was carried out using the PSI-BLAST and DELTA-BLAST programs from the NCBI-BLAST package, v.2.6.0. The search was performed with five iterations, resulting in 6362 unique PolX sequences found in 6239 genomes. To construct a non-redundant, representative sequence set for the phylogenetic and sequence analysis, the PolX sequences were clustered using the UCLUST 4.2 software (44) with the sequence identity threshold of 95%, resulting in a collection of 2935 unique sequences from 2846 genomes. The sequences were aligned by the MAFFT v.7.450 software (–genafpair –maxiterate 1000) (45). The resulting alignment is presented in Supplementary Dataset (2935_polX.fasta).

To identify domains and functionally important residues, the sequences of human Polβ, *T. thermophilus* PolX and *B. subtilis* PolX were used as references. The columns of alignment corresponding to positions of interest were collected and analyzed in R. To estimate the lengths of PolX domains, the parts of the alignment corresponding to Polβ residues 178–279 for palm and 272–314 for fingers were extracted and analyzed using R packages biostrings, seqinr, ape, protr and tidyr. Structural modeling was performed with the colab version of AlphaFold2, visualizations were performed with PyMol (https://pymol.org/) and VMD programs (46,47).

**Figure 2.**
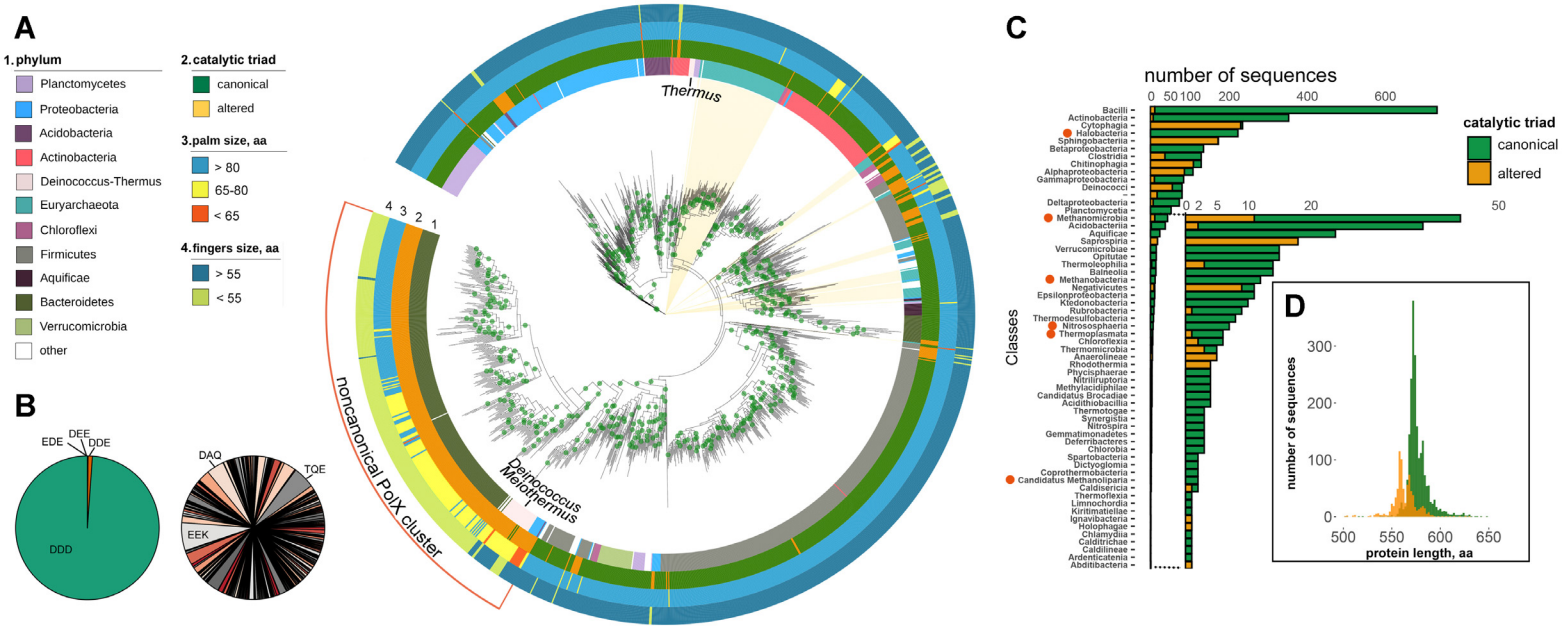
The diversity of prokaryotic PolXs. (**A**) The phylogenetic tree of PolX proteins based on the multiple alignment 1433 non-redundant PolX sequences. Positions of archaeal PolXs on the tree are marked by light ochre sectors. The features of PolX proteins are annotated as follows (from the inner to the outer rings): 1, phylum; 2, the status of the catalytic triad in the palm domain (canonical, green; altered, ochre); 3, the size of the palm domain (blue, ≥80 residues; orange, 65–80 residues; red, <65 residues); 4, the size of the fingers domain (dark blue, ≥35 residues; green, <35 residues). The main cluster of noncanonical PolXs is indicated. The green dots on the nodes correspond to bootstrap values of 98-100. (**B**) Distribution of catalytic triad residues in canonical PolXs (left) and altered PolXs (right, three most abundant variants are indicated; see Supplementary Table S1 for altered triad frequencies). (**C**) Distribution of canonical and altered PolX sequences among prokaryotic classes (archaeal classes are indicated with red circles). (**D**) Distribution of the lengths of canonical and altered PolXs.

The phylogenetic tree shown in Figure 2 was constructed from an alignment of 1433 unique PolX sequences obtained by further clustering of the non-redundant collection of 2935 sequences with MMseqs2 Version: 13.45111, using the sequence identity threshold level of 80% (46–48). Positions containing >50% gaps were removed from the alignment with TrimAl ver.1.2 (49). The tree was built using the IQTREE version 2.1.4-beta and visualized with iTol (https://itol.embl.de/shared/p5XbdEu6WXeO) (50). Local support values were calculated by the IQTREE ultrafast bootstrap test with 1000 replicates (51). The alignment of 1433 PolXs and the resulting tree in a machine-readable format can be found in Supplementary Dataset (1433_polX_aln_trim.fasta and 1433_polX_tree.contree).

For analysis of co-occurrence of PolX, LigD and Ku in bacterial genomes (Figure 5A), fully sequenced bacterial genomes were fetched from the NCBI FTP site in November 2021 (“complete” genome or chromosome status, 24973 genomes in total). Search for genes encoding PolX, LigD and Ku was performed with the complete genome sets with deltablast 2.12.0 (-num_iterations = 5 -evalue = 0.005 -max_target_seqs 100000), using amino acid sequences of PolXs (*B. subtilis* P94544, *T. thermophilus* A0A3P4ARX8, *D. radiodurans* Q9RX48), the Ku protein (*Mycobacterium tuberculosis* P9WKD9, *Pseudomonas aeruginosa* Q9I1W5, *B. subtilis* O34859), and individual domains of LigD: ligase LIG (*P. aeruginosa* Q9I1×7|219-521, *M. tuberculosis* P9WNV2|460-757, *B. subtilis* O34398|5-310), polymerase POL (*B. subtilis* O34398|325-564, *P. aeruginosa* Q9I1X7|549-793, *M. tuberculosis* P9WNV2|9-261), and phosphoesterase (*M. tuberculosis* P9WNV2|297-446, *P. aeruginosa* Q9I1X7|7-162) as queries. Rpsblast v. 2.12.0 was used to find conserved domains in the resulting sequences and those containing CDD 213991 or 214688 (PolX), CDD 224192 (Ku), CDD 274293 or 274295 (LIG), CDD 240135 or 240132 or 240133 or 240134 or 240136 (POL), and CDD 131824 (phosphoesterase) were selected. The corresponding genomes were assigned as containing these proteins or domains. Further analysis was performed with custom R scripts. To build the phylogenetic tree shown in Figure 5B, 2826 non-redundant representative genomes from various bacterial lineages were sampled from the full collection of genomic sequences using the tool for getting clusters of similar genomes (https://microbiome.wordpress.com/research/redundancy/), using a genomic similarity score (GSS) of <95 (52). These genomes contained 452 PolX sequences. To calculate statistical significance of co-occurrence of the NHEJ proteins and PolXs in different bacterial phyla, contingency tables containing the numbers of genomes with different NHEJ status (no NHEJ, NHEJ+Nuc or NHEJ-Nuc) and PolX status (no PolX, altered or canonical PolX) were prepared for the non-redundant collection of 2826 full genomes or for individual phyla from this collection. The resulting tables were used to perform Pearsоn Chi-square test of independence implemented in R.

For analysis of genomic neighborhoods of PolX genes, only full genomes corresponding to the non-redundant collection of PolXs were selected. In total, we identified 2620 full genomes corresponding to 1249 non-redundant PolX sequences (1074 canonical and 175 altered). For each genome, genes co-directional with the PolX gene within 5 upstream and 5 downstream genes were selected, located within 300 bp from each other. The resulting databases were analyzed in R, and the number of genomes containing different Pfam families were calculated separately for canonical and altered PolXs from bacteria from various taxonomical classes. Classes with less than four genomes and Pfam families with less than 10% frequency in the class were excluded from the resulting table (Supplementary Table S4).

### Sequencing of PolX genes

To amplify PolX genes, genomic DNA from *Flectobacillus major*, *Belliella baltica* and *Pedobacter insulae* was used in PCR reactions with oligonucleotide pairs Flema_F 5′-CCGACGCAGTACGGGTTATT, Flema_R 5′-GCCTGCTGAATCTTGCCCTA; Belba_F 5′-CAGCAAAGTCAACTTGAGCTAGA, Belba_R 5′-ATCCCAACTCCATTCAGCGG; and Pedin_F 5′-TGTTCGCTCATAGGCGTTGT, Pedin_R 5′-CACTGCGCCGTATCTATCCA, respectively. The PCR fragments were purified from agarose gel and sequenced with the same oligonucleotide primers and additional primers Flema_in 5′-TTCCGACAGAGTATTCTTGC, Belba_in 5′-CAGGGTATGTTTTCCATCAC, and Pedin_in 5′-TCGCAAATTCCCAGATACTC in a Sanger sequencing facility. The resulting sequences were compared to nucleotide sequences corresponding to PolX proteins with GenBank IDs WP_026994533.1, WP_041779305.1 and WP_090994746.1 from genomes with GenBank IDs GCA_000427405.1, GCA_000265405.1 and GCA_900113525.1, respectively.

### Cloning, expression and purification of PolXs from *Deinococci* and *B. subtilis*

The PolX gene of *D. radiodurans* (GenBank ID WP 010887112.1) was amplified from genomic DNA of *D. radiodurans* using primers Dra_NdeI 5′ CATTAGCATATGACCCTGCCGCCCGACGC and Dra_SalI 5′-TATCTAGTCGACTTATGCACGGTCCGCCGGGCCG and cloned into pET28a between the sites of NdeI and SalI. The PolX gene of *Deinococcus gobiensis* (GenBank ID AFD24201.1) was codon-optimized and obtained by custom gene synthesis from IDT (using two overlapping gBlocks Gene Fragments). The synthetic gene was amplified using primers Dgo_F 5′ CTTTAAGAAGGAGATATACATATGCATCAC and Dgo_R 5′ CTTGTCGACGGAGCTCGAATTCGTCATTACCCAGCGTTTGCACGTGC, and cloned in the same way. The PolX gene of *B. subtilis* (GenBank ID WP_063335829.1) was amplified from genomic DNA of *B. subtilis* using primers Bsu_NdeI 5′ cggcagccatATGCATAAAAAAGATATTATCCGGC and Bsu_SalI 5′-gcttgTCGACTTAATCGTTGCGCTTCAGAAAT and cloned in the same way. Mutations in the polymerase catalytic triad in *D. radiodurans* PolX (E199A/E234A) and *B. subtilis* PolX (D193A) and in the PHP active site in *D. radiodurans* PolX (H332A/H334A) were introduced in the expression plasmids by Kunkel mutagenesis. All plasmid clones were verified by Sanger sequencing.

*E. coli* BL21 (DE3) cells were transformed with the PolX plasmids and several colonies were inoculated into 1 L of LB medium with 100 μg/ml ampicillin, grown at 37°C until OD_600_ ∼0.5 and chilled on ice for 30 min. IPTG was added to 0.05 mM and the culture was grown at 16°C overnight. The cells were precipitated by centrifugation and resuspended in buffer containing 30 mM HEPES-KOH pH 7.4, 1 M KCl, 10 mM K_2_HPO4, 5% glycerol, 4 mM β-mercaptoethanol, 2 mM PMSF, and lysed with a high pressure homogenizer at 4°C. The lysate was cleared by centrifugation at 15 000 rpm on a Hitachi CR22N centrifuge at 4°C and loaded onto a 1 ml Ni-Sepharose column (GE Healthcare), equilibrated with buffer containing 30 mM HEPES–KOH pH 7.4, 1 M KCl, 10 mM K_2_HPO_4_, 5% glycerol. The column was washed with the same buffer containing 20 mM imidazole, and PolX was eluted by the same buffer containing 300 mM imidazole. Fractions containing PolX were pulled, diluted ten times by the same buffer without KCl and loaded onto a 1 ml Heparin-Sepharose column (GE Healthcare), equilibrated with the same buffer containing 80 mM KCl. PolX was eluted by a KCl gradient from 80 to 800 mM (40 ml). Fractions containing PolX were pulled, diluted 5 times by the same buffer without KCl and loaded onto a 1 ml MonoQ column (GE Healthcare), equilibrated with the same buffer with 50 mM KCl. PolX was eluted by a KCl gradient from 80 to 800 mM, fractions containing PolX were pulled, aliquoted, frozen in liquid nitrogen and stored at -80°C. The purity of the samples was at least 98% based on SDS-PAGE analysis (Supplementary Figure S3A). The stability of the *B. subtilis* and *D. radiodurans* Pol Xs (1.6 and 0.3 mg/ml respectively) in the phosphate buffer pH 7.5 were measured by thermal unfolding using a Tycho NT.6 instrument (NanoTemper Technologies, Germany). Circular dichroism spectra for the same PolX preparations were measured on a Chiroscan CD spectrometer (Applied Photophysics) with 2 nM bandwidth at 22°C (Supplementary Figure S3B).

### *In vitro* analysis of PolX activities

To obtain DNA substrates for analysis of DNA polymerase and exonuclease activities of PolXs (Figure 4B), 5′-P^32^-labeled primer (400 nM) and unlabeled template (440 nM) oligonucleotides were annealed in 100 mM KCl (5 min incubation at 70°C followed by cooling down to 25 °C at ∼1°C/min). For assembly of gapped substrates, a third 5′-P or 5′-OH downstream nontemplate oligonucleotide was added (440 nM).

PolXs were first incubated for 5 min at 30°C in the reaction buffer containing 30 mM HEPES-KOH pH 7.4, 50 mM KCl, 1 mM DTT, 0.1 mg/ml BSA and 1 mM EDTA, to remove residual divalent cations. Then *D. radiodurans* PolX, *B. subtilis* PolX (20 nM final concentration) or *D. gobiensis* PolX (50 nM) were added to DNA substrates (20 nM) in the same buffer lacking divalent cations or containing 11 mM MgCl_2_ or 2 mM MnCl_2_. In experiments shown in Supplementary Figure S4A-D, 1 μM of PolXs was incubated with 5 nM of DNA substrates. 200 μM or 10 μM of each dNTP where added and the reactions were stopped after 0, 10, 30, 90 min by adding an equal volume of 98% formamide and 50 mM EDTA. To determine optimal divalent metal concentration, the reactions were performed in the same conditions for 30 min in the presence of 100 μM EDTA and varying MgCl_2_ or MnCl_2_ concentrations (0, 0.25 0.5, 1, 3, 5, 10, 20 or 50 mM). In experiments shown in Supplementary Figure S4F (the reaction conditions corresponding to ref. (31)), the reaction mixture contained 50 mM Tris-HCl pH 7.5, 2 mM DTT, 100 μM of each dNTP, 50 nM *D. radiodurans* PolX and 50 nM 5′-P^32^-labeled DNA substrate. The reactions were initiated by adding a mixture of MgCl_2_ (5 mM) or MnCl_2_ (2 mM) and excess of unlabeled DNA substrate (5 μM) and stopped after 0, 10, 30, 90 min. The samples were incubated for 3 min at 98°C and analyzed by 23% denaturing PAGE followed by phosphorimaging with a Typhoon 9500 scanner (GE Healthcare).

## RESULTS

### Phylogenetic and structural diversity of prokaryotic PolXs

To analyze the diversity of prokaryotic PolXs, we searched for PolX sequences in the NCBI Refseq genomic database based on homology with previously studied bacterial PolXs. In total, we identified 6362 PolX sequences in about 13% of bacterial and 31% of archaeal full genomes. For further analysis, we used a non-redundant collection of PolX sequences with <95% identity that contained 2935 unique polymerases, 2639 from bacteria and 296 from archaea (Figure 2A). The number of polymerases found in different Bacterial and Archaeal classes was highly uneven, partially as a result of highly different numbers of sequenced genomes in each phylum (Figure 2C). Class Bacilli (734 sequences), containing a large number of sequenced genomes of important human pathogens and cohabitants, was most abundant among Bacteria, other abundant classes included bacteria from the human microbiome. Class Halobacteria/Haloarchaea (224 sequences), containing common laboratory models, was most abundant among Archaea; 288 out of 296 archaeal sequences belonged to the *Euryarchaeota* phylum.

To identify key structural and functional motifs of PolXs, we performed multiple sequence alignment of the PolX sequences and defined the boundaries of individual protein domains using *T. thermophilus*, *B. subtilis* and human PolXs as references (see Materials and Methods) (Supplementary Dataset). The mean length for all prokaryotic PolXs is 573.1 ± 23.7 amino acid residues and the median is 573 residues; the length of 10 sequences is <400 and the length of 9 sequences is >700 residues, indicating that the collection largely includes full-sized PolXs (Figure 2D). The overall domain arrangement is well conserved in prokaryotic PolXs, and the majority of them contain five structurally distinct domains from the N- to C-end: 8 kDa dRP-lyase, thumb, palm, fingers, and 3′-5′ exonuclease PHP domains (Figure 1B). However, we revealed significant variations in the structure of DNA polymerase domains involved in catalysis, which are described in detail below.

Analysis of the maximum likelihood phylogenetic tree built from the amino acid alignment of PolXs showed that some bacterial phyla are split and interleaved in the PolX tree (Figure 2A). In particular, a substantial number of PolX sequences from *Deinococcus*, *Proteobacteria* and *Firmicutes* are closely related to PolXs from *Bacteroidetes*, while the rest PolXs from these phyla form monophyletic groups or are related to PolXs from other phyla (Figure 2A). These data indicate likely horizontal transfer of PolX genes between bacterial phyla. The largest group of PolXs found in archaea have a monophyletic origin and are distantly related to bacterial PolXs from *Actinobacteria* (Figure 2A). In addition, several smaller groups of archaeal PolXs are found in other branches of the PolX tree and are interleaved with bacterial sequences. This indicates that some archaea could have obtained the PolX gene *via* horizontal transfer from bacteria, which is not uncommon in archaea in general and in Haloarchaea in particular (53,54).

### Noncanonical PolXs have an altered catalytic site in the palm domain

**Figure 3.**
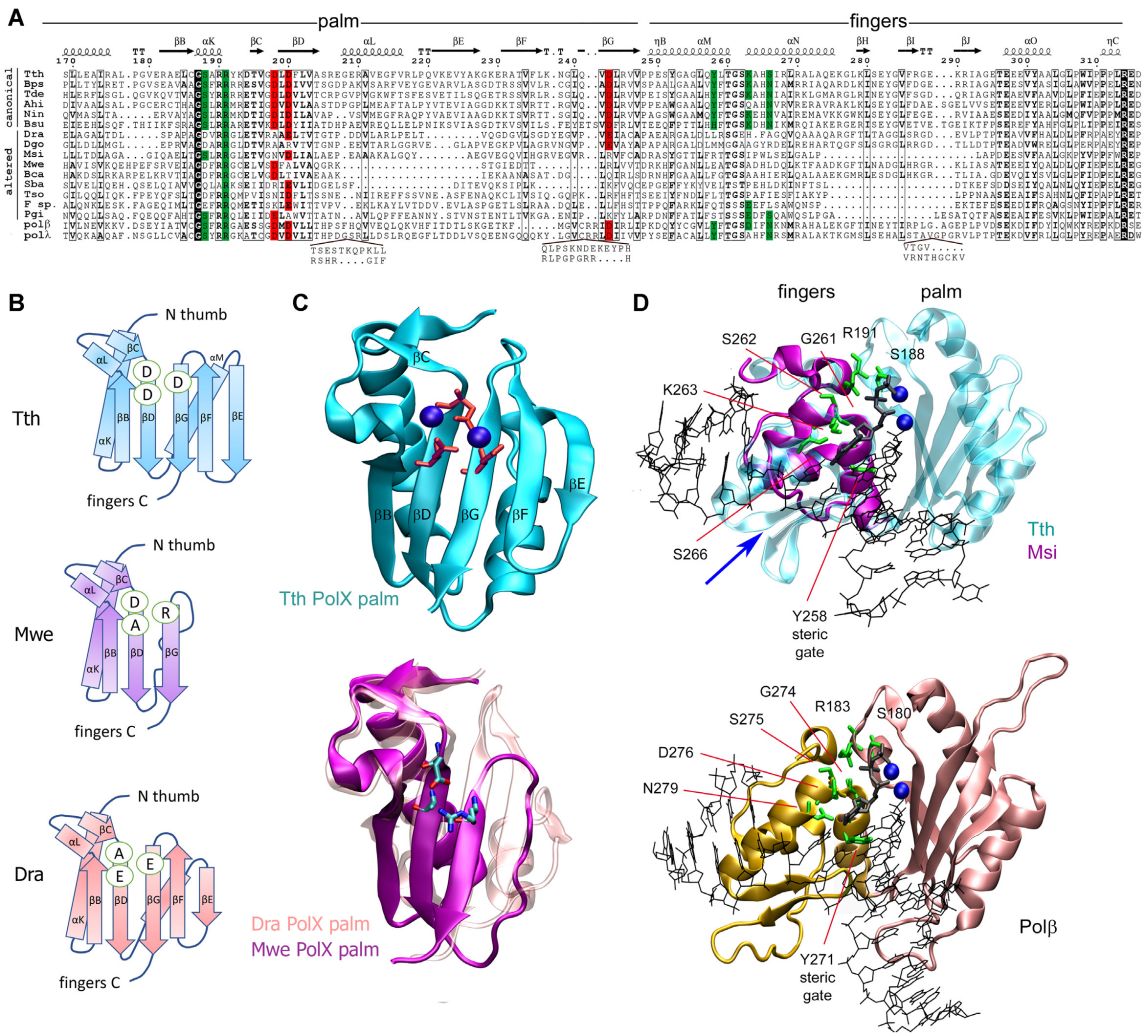
The catalytic site of canonical and altered PolXs. (**A**) Alignment of the sequences of the palm and fingers domains in PolXs. The catalytic triad residues (aspartate or glutamate) are shown in red, the conserved dNTP binding residues are green; similar residues (similarity score >0.7 in the non-redundant collection of PolXs) are shown in bold, absolutely conserved residues are shown in black. The abbreviations of the species names are as follows: Tth, *Thermus thermophilus*; Bps, *Burkholderia pseudomallei*; Tde, *Thiohalomonas denitrificans*; Ahi, *Actinomadura hibisca*; Nin, *Nonomuraea indica*; Bsu, *Bacillus subtilis*; Dra – *Deinococcus radiodurans*; Dgo – *Deinococcus gobiensis*; Msi – *Meiothermus silvanus*; Mwe - *Mesorhizobium wenxiniae*, Bca - *Bradyrhizobium canariense*, Sba - *Sphingobacteriaceae bacterium*, Tso - *Taibaiella soli*, F. sp. - *Flavisolibacter sp*. X7X, polβ and polλ - human Polβ and Polλ. The protein secondary structure is shown above the alignment for *T. thermophilus* PolX. Amino acid numbering for *T. thermophilus* PolX is shown above and below the alignment. Sequence alignments of Dra and Dgo PolXs were manually curated according to the Dra PolX structure. (**B**) Schematic representation of the palm domain topology for *T. thermophilus* (top), *M. wenxiniae* (middle) and *D. radiodurans* (bottom) PolXs. The catalytic triad residues are indicated in ovals. (**С**) (*Top*) The structure of the palm domain of *T. thermophilus* PolX (PDB: 3AUO). The catalytic triad residues are shown in red as stick models, Mg^2+^ cations are shown as blue spheres. (*Bottom*) Superimposition of the structure of the palm domain of *D. radiodurans* PolX (pink, PDB: 2W9M) and a modeled structure of the palm domain of *M. wenxiniae* PolX (magenta). (**D**) Structures of the fingers domain in prokaryotic PolXs and human Polβ. (*Top*) Superimposition of the structures of *T. thermophilus* PolX (fingers and palm domains, turquoise) in complex with gapped DNA (PDB: 3AU0) and of the fingers domain of *M. silvanus* PolX (violet), based on structural modeling; the position of the deleted region is indicated with a blue arrow. (*Bottom*) Structure of human Polβ with gapped DNA (PDB: 1BPY). Mg^2+^ ions bound in the active site of the palm domain are shown as blue spheres, the incoming dNTP is black. Positions of functionally important residues (green) in the fingers domain are indicated.

The palm domain of PolX belongs to the Polβ-like nucleotidyltransferase superfamily (21) and has an αβαββαβββ topology, in which five β strands form one mixed β sheet containing three conserved acidic residues (usually three aspartates), involved in the binding of catalytic metal ions, in adjacent β strands (Figure 3A,B). Most prokaryotic PolXs (2164; 72.5% in our dataset) contain three acidic residues (aspartate or rarely glutamate) in corresponding positions and probably retain the DNA polymerase activity (Figure 2B). We classify these PolXs as canonical polymerases. Surprisingly, besides the prevailing PolX variants with the canonical catalytic triad, we identified a group of polymerases (809; 27.5%) that partially or totally lack the conserved acidic residues in the polymerase active site. We classify these polymerases as altered or noncanonical PolXs (Figures 2 and 3A, B). Variations of the active site motif in the noncanonical polymerases include substitutions of one, two or all three aspartate residues with non-charged or even positively charged residues, and comprise 302 unique variants (Figure 2B, Supplementary Table S1, Figure 3A). The substitutions include, but are not limited to, lysine, arginine, threonine, valine, alanine etc., and in the majority of the cases substantially change the electrostatic environment of the active site region. Therefore, initial inactivation of the polymerase site in noncanonical polymerases was likely followed by additional substitutions in the non-functional active site thus generating many triad variants.

Noncanonical polymerases with altered catalytic triads are found in different bacterial phyla but most of them form a single cluster on the phylogenetic tree and likely have monophyletic origin (Figure 2A). This cluster is mainly formed by PolXs from the *Bacteroidetes* and *Deinococcus-Thermus* phyla, and most PolXs from these phyla are noncanonical. Interestingly, in the class *Deinococci*, which includes previously studied PolXs from *D. radiodurans* and *T. thermophilus*, altered polymerases belong mostly to the genera *Meiothermus* and *Deinococcus*, while canonical polymerases belong mostly to the genus *Thermus*. The main cluster of noncanonical polymerases also contains several PolXs from other phyla including *Proteobacteria* and two polymerases of *Acidobacteria* and *Rhodothermaeota*. The phylogenetic relatedness of the majority of noncanonical polymerases suggests their common evolutionary origin while the presence of related PolX variants in unrelated bacterial lineages indicates their horizontal transfer, similarly to canonical PolXs (19).

In addition, there are several smaller groups of noncanonical PolXs, separated from the main cluster, found in bacteria and archaea (Figure 2A). In bacteria, many of these polymerases are found in Firmicutes. Among Archaea, 11 from 12 altered polymerases (from the non-redundant collection of PolX sequences) belong to the class *Methanomicrobia* and also form a separate clade from the majority of noncanonical PolXs. This suggests that noncanonical PolXs with substitutions in the polymerase active site have likely appeared several independent times in the evolution. Further research is needed to fully understand the origin and evolution of noncanonical PolX, including analysis of additional PolX sequences from many under-represented prokaryotic phyla.

In addition to substitutions of the catalytic residues, many noncanonical PolXs bear deletions in the palm domain (Figures 2A and 3A). The palm domain length is quite constant in canonical polymerases, with the mean domain size of 80.93 residues (95% CI 80.85–81.01) (Supplementary Figure S1). In comparison, the average protein length of noncanonical PolXs is shifted to smaller values in comparison with canonical polymerases (Figure 2D). A particular group of noncanonical PolXs containing the shortest palm domains with deletions of 13-26 amino acids (mean domain size of 56.6 residues [95% CI 56.3–57.0]) is clustered together on the PolX tree (red sector in the main cluster of noncanonical PolXs in the palm ring in Figure 2A, highlighted with a dashed line in Supplementary Figure S1). Most noncanonical polymerases with truncated palm domains (<77 amino acids) belong to classes *Alphaproteobacteria* (86, the group with the shortest palm variants, 54% of truncated PolXs), *Deinococci* (23, 14.5%), and *Saprospiria* (13, 8.2%) (Figure 2A).

In the two solved structures of prokaryotic PolXs from *T. thermophilus* and *D. radiodurans*, the palm domain adopts the classical Polβ nucleotidyltransferase fold (Figure 3B, C) (28,32,35,55–57). The overall organization of the *T. thermophilus* PolX active site is very similar to human Pol β, while *D. radiodurans* PolX has significant differences and contains an altered catalytic triad, AEE (56). Furthermore, *D. radiodurans* PolX has a deletion of 7 amino acids in comparison with *T. thermophilus* PolX, which results in significant shortening of the β strand E (Figure 3B and C). A structural model of *Mesorhizobium wenxiniae* PolX containing a DAR triad in the catalytic site reveals an even more drastic deletion (26 residues) in the palm domain, in particular of the α helix M and β strands E and F (Figure 3B and C).

It should be noted that the amino acid context of the substituted triad residues is well conserved in noncanonical PolXs, which allows their unambiguous identification in most sequences (Figure 3A, Supplementary Dataset). Furthermore, substitutions and deletions in noncanonical PolXs are unlikely to represent sequencing artifacts, since they are found specifically in the palm and fingers domains, but not in other parts of PolX (see below). Moreover, most noncanonical PolXs are clustered on the phylogenetic tree, suggesting their evolutionary relationship (Figure 2A). In addition, we performed Sanger sequencing of noncanonical PolXs for several bacterial species from our laboratory collection including *Flectobacillus major*, *Belliella baltica* and *Pedobacter insulae*, all with altered catalytic triads and truncated domains. In all cases, the reported changes were present in the sequences, confirming the correctness of PolX sequences deposited in the genomic database.

### Changes in the dNTP binding site in noncanonical PolXs

The fingers domain plays the key role in the binding of dNTP substrates during catalysis. The dNTP binding pocket in PolX polymerases is formed by two conserved motifs in the palm and fingers domains (187-GSARR-191 and 258-YLTGSKAHS-266 in *T. thermophilus* PolX; 179-GSFRR-183 and 271-YFTGSDIFN-279 in human Polβ) (Figure 3B and D). Residues D276 and N279 (Polβ numbering) form Van der Waals and hydrogen bonds with the dNTP base, respectively; residue Y271 serves as a ‘steric gate’ responsible for dNTP/rNTP discrimination (Figure 3D, Supplementary Table S2) (58–64). Residues 274-GS-275 form a characteristic *cis* peptide bond in Polβ and TdT, which stabilizes the two α-helixes containing residues involved in dNTP binding, and interact with residue R183 from the palm domain (65–67). Substitutions of most of these residues in Polβ were shown to affect its activity and fidelity by changing the interactions with the incoming nucleotide (58–64).

The importance of the dNTP binding residues for catalysis was also confirmed in studies of prokaryotic PolXs. In particular, substitutions of residues corresponding to Polβ N279 in PolXs from *B. subtilis* and *T. thermophilus* (N263 and S266, respectively) significantly affected nucleotide incorporation (32,55,68). Interestingly, both *B. subtilis* and *T. thermophilus* PolXs contain a lysine residue in place of D276 in Polβ (K260 and K273, respectively). This residue was proposed to stabilize the incoming nucleotide, and its substitutions lowered the affinity of prokaryotic PolXs to dNTP substrates (32,68). The presence of a lysine at this position can explain the ability of *T. thermophilus* PolX to form a stable complex with dNTP in the absence of DNA and may favor an unusual mechanism of nucleotide incorporation, in which the binding of dNTP precedes the binding of DNA (32). Interestingly, a basic residue in this position is also present in TdT and Polμ but not in Polλ and viral ASFV PolX, all of which can also bind dNTP in the absence of DNA and are capable of non-templated DNA synthesis, suggesting that this residue is not the sole determinant for such interactions (20,69–72).

Our analysis demonstrated that the asparagine residue corresponding to N279 in Polβ is highly conserved in canonical PolXs (92.87% N) and is substituted by a hydrophobic residue in almost all altered polymerases (Supplementary Table S2). The basic residue in position corresponding to D276 of Polβ is also conserved among canonical polymerases (K 84.8%, R 6.8%), suggesting that most of them use a similar mechanism for dNTP binding. In contrast, this residue is not conserved in noncanonical polymerases (K 7.6%, R 4.2%), and is often substituted with E (24.1%) (including PolX from *D. radiodurans*), A (21.5%) or P (14.9%) (Supplementary Table S2). Furthermore, residues corresponding to the *cis*-peptide bond motif 274-GS-275 in the fingers and residue R183 in the palm domain in Polβ are highly conserved in canonical prokaryotic PolXs (97.6% and 100% respectively), suggesting that these polymerases preserve a functional conformation of the fingers domain during catalysis. In contrast, the GS motif is much less conserved in noncanonical polymerases (42%) and is often substituted with GN, AS or AA. Finally, the steric gate motif is found in most canonical PolXs (YF in 54.3% and HF in 40% of sequences) but is not at all conserved in altered polymerases (Supplementary Table S2). Together, the absence of conservation of key residues of the dNTP binding site in the noncanonical polymerases suggests that they have an impaired ability to coordinate incoming nucleotides in the active site.

Many altered polymerases also have a truncated fingers domain in comparison to canonical PolXs (Figure 2A, Supplementary Figure S1). The deletions can remove up to three successive β strands and a part of the α-helix, as revealed by structural modeling of PolXs from *Meiothermus silvanus* and its relatives (Figure 3D). In the complex of *T. thermophilus* PolX and Polβ, this part of the fingers domain interacts with the template DNA strand, and its absence in noncanonical polymerases may potentially affect their interactions with DNA (Figure 3D). Truncation of the fingers domain often accompanies deletions in the palm domain. In total, we revealed 55 such ‘double-truncated’ (palm < 77 amino acid residues, fingers < 55 residues) polymerases among the 2935 non-redundant PolX sequences. All of them also have altered catalytic triads. The double-truncated polymerases are abundant in the phyla *Bacteroidetes* (29 PolX variants) and *Deinococcus-Thermus* (17 PolX variants).

Overall, these results indicate that the noncanonical polymerases have a degraded active site with multiple substitutions and deletions in both the palm and fingers domains involved in catalysis.

### High conservation of the nuclease domains in noncanonical PolXs

The C-terminal exonuclease PHP domain is specific for prokaryotic PolXs and is absent in eukaryotic PolXs (Figure 1B). The PHP domain is also found as an additional domain in prokaryotic C family DNA polymerases and as a stand-alone domain in histidinol phosphatases (22). In replicative C family DNA polymerases, it can be inactive due to substitutions of catalytic residues (Pol III in *E. coli*) or active (Pol C in *B. subtilis, M. tuberculosis, T. thermophilus*), thus providing the proofreading activity during DNA replication (73–76).

**Figure 4.**
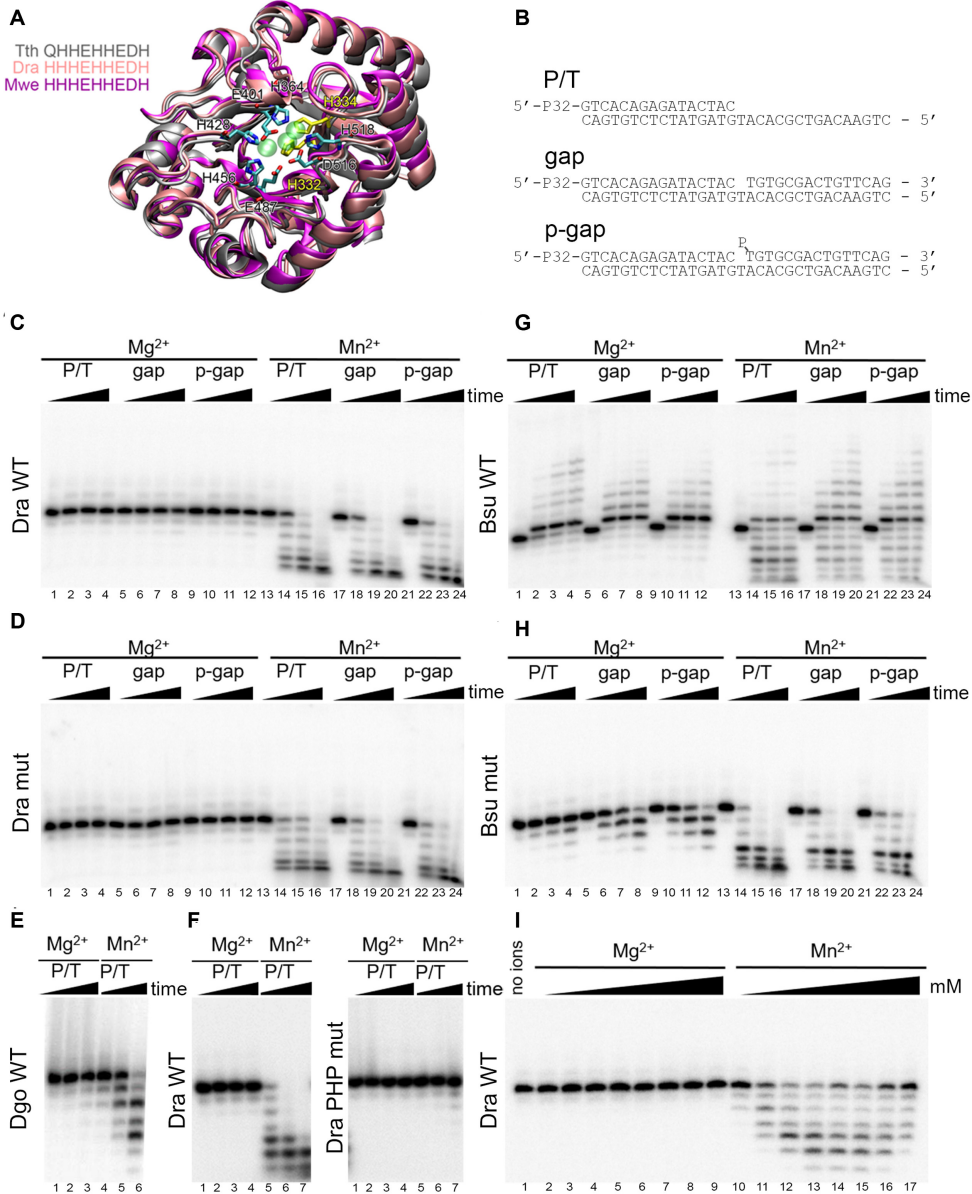
Analysis of the catalytic activities of noncanonical PolXs. (**A**) Structure of the PHP domain of bacterial PolXs. Superposition of the structures of PHP domains of *T. thermophilus* PolX (gray, PDB: 3AUO), *D. radiodurans* PolX (pink, PDB: 2W9M) and a modeled structure of *M. wenxiniae* PolX (magenta) is shown. Zn^2+^ ions from the crystal structure of *D. radiodurans* PolX are shown as semitransparent green spheres. The active site residues are shown as stick models, the residues mutated in this study in *D. radiodurans* PolX are shown in yellow. The C-terminal α-helix is not shown for clarity. (**B**) Schemes of the DNA substrates: primer-template (P/T) and gapped substrates with 5′-OH or 5′-phosphorylated downstream oligonucleotide (gap and p-gap, respectively) used for analysis of PolX activities. (**C**, **D**) Analysis of the activities of *D. radiodurans* PolX in the presence of 200 μM dNTP substrates and 11 mM Mg^2+^ or 2 mM Mn^2+^ cations. The reactions were performed with 20 nM of wild-type PolX from *D. radiodurans* (**C**) or its palm domain mutant with substitutions in the polymerase active site (**D**) at 30°C for 0, 10, 30, 90 min. (**E**) Analysis of the activity of wild-type PolX from *D. gobiensis* on the P/T substrate in the same reaction conditions. (**F**) Comparison of the activities of wild-type *D. radiodurans* PolX and its PHP mutant with substitutions in the exonuclease site in the same reaction conditions. (**G**,**H**) Analysis of the activities of wild-type PolX from *B. subtilis* (**G**) and its mutant with a single substitution in the catalytic triad (**H**) in the same reaction conditions. (**I**) Determination of optimal divalent metal ion concentration for wild-type PolX from *D. radiodurans*. The concentrations of Mg^2+^ and Mn^2+^ were varied between 0.25 and 50 mM in the presence of 200 μM dNTPs. For all experiments, representative gels from two-three independent replicates are shown.

The PHP domain of prokaryotic PolXs was reported to have the 3′-5′ exonuclease, AP-endonuclease, 3′-phosphodiesterase, and 3′-phosphatase activities (23,25,33,68,77). The 3′-5′ exonuclease activity of prokaryotic PolXs was observed on single-stranded DNA as well as on primer/template substrates and was shown to be modulated by the secondary structure of the DNA substrate (23–25,34). All catalytic activities of the PHP domain depend on the same metal-chelating (Mn^2+^-dependent) active site, which is formed by four motifs with nine conserved residues (the HHHEHHEDH consensus) that coordinate divalent metal cofactors (Figure 4A, Supplementary Figure S2). Structural comparisons of the PHP domains from the canonical *T. thermophilus* and altered *D. radiodurans* PolXs and a modeled structure of *M. wenxiniae* PolX revealed almost no differences in the positions of the active site residues, which similarly coordinate two or three divalent cations (Figure 4A) (32,56).

The PHP domains of the majority of bacterial and archaeal PolXs included in our analysis retain 8–9 conserved residues in the active site (Supplementary Figure S2). In both types of PolXs, the most abundant motif is the HHHEHHEDH consensus (88% in canonical and 98% in altered polymerases, including *D. radiodurans* PolX, among the 2935 non-redundant PolX sequences). Variations of this motif are more common in canonical polymerases and include HHPERHEDQ (2.7%), HHRERHEDC (2.3%), QHHEHHEDH (1.3%, including *T. thermophilus* PolX) and HHRERHEDM (1%). Together, the data indicate that the active site of the PHP domain is extremely conserved in prokaryotic PolXs, suggesting that its functional activities are important in both types of polymerases.

In addition to the conserved C-terminal PHP domain, most prokaryotic PolXs also contain an intact N-terminal (8 kDa) domain (Figure 1B). In eukaryotic PolXs, this domain together with thumb participates in the binding of gapped DNA substrates and contains residues responsible for the dRP-lyase activity (3). In Polβ, it plays the key role in the processing of gapped DNA and directly recognizes the 5′-P or 5′-dRP groups of a gap/nick (67,78). The 8 kDa and thumb domains are well conserved in both canonical and noncanonical PolXs, with the full-length 8 kDa domain found in 95.3% of all sequences (Supplementary Dataset), suggesting that most of them may retain the dRP-lyase activity potentially important for their functions in DNA repair.

### Functional analysis of noncanonical PolXs from *Deinococcus* species

Substitutions in the catalytic triad and changes in other parts of the palm and fingers domains suggest a loss of the DNA polymerization activity in noncanonical PolXs. Indeed, the aspartate triad is essential for the metal ion coordination and catalytic activity in the Polβ superfamily of nucleotidyltransferases (35,36,42,79,80). Similarly, even single substitutions in the catalytic triad in other polymerases dramatically decrease the rate of DNA polymerization (81). Surprisingly, a template-dependent polymerase activity was reported previously for recombinant PolX from *D. radiodurans*, a noncanonical PolX with an AEE triad, containing alanine and two glutamates instead of aspartates (31). However, no metal ions are bound in the active site in the published structure of *D. radiodurans* PolX, indicating that the substitutions impair catalytic metal binding by this PolX (Figures 1E and 3C) (56).

To study the spectrum of activities of noncanonical PolXs, we purified and analyzed recombinant PolX polymerases from *D. radiodurans* and *D. gobiensis*. Similarly to *D. radiodurans* PolX, the latter polymerase contains a noncanonical triad (ARE), in which all three aspartates are substituted with other residues (Figure 3A). While the wild-type *D. radiodurans* PolX gene was successfully expressed in *E. coli*, a codon-optimized version of the *D. gobiensis* PolX gene was designed to increase its expression (see Materials and Methods). To avoid admixtures of cellular polymerases or nucleases, we performed three chromatographic steps during PolX purification, including Ni^2+^-chelating, heparin affinity and anion exchange chromatography and resulting in highly pure PolX preparations (Supplementary Figure S3A). In addition to the wild-type enzymes, we obtained a mutant variant of *D. radiodurans* PolX with alanine substitutions of the two glutamate residues in its active site (E199A/E234A). As a control canonical polymerase, we also expressed and purified *B. subtilis* PolX and its mutant variant with a single amino acid substitution in the catalytic triad (D203A). To confirm that noncanonical *D. radiodurans* PolX has a native conformation, we measured its circular dichroism spectrum and found that it is highly similar to that of *B. subtilis* PolX and to the predicted spectrum based on the content of α and β structures in the *D. radiodurans* PolX structure (Supplementary Figure S3B) (82). Furthermore, measurement of the denaturation temperatures (Td) for these polymerases demonstrated that *D. radiodurans* PolX is even more thermoresistant than *B. subtilis* PolX (Td of 83 and 61.6°C, respectively), suggesting that it forms a stable structure.

The activity of *D. radiodurans*, *D. gobiensis* and *B. subtilis* PolXs was tested on primer-template or gapped DNA substrates (Figure 4B) in the presence of dNTPs and Mg^2+^ or Mn^2+^ ions. It was found that noncanonical *D. radiodurans* PolX is unable to extend the primer in the presence of Mg^2+^ on any of the tested templates at either low or high polymerase (20 nM or 1 μM) or dNTP (10 or 200 μM) concentrations (Figure 4C, lanes 1–12; Supplementary Figure S4A, lanes 1-12; Supplementary Figure S4E, lanes 1-12). Not surprisingly, mutant *D. radiodurans* PolX with alanine substitutions in the active site was also inactive in these assays (Figure 4C, lanes 1–12; Supplementary Figure S4B, lanes 1–12). Similarly, *D. gobiensis* PolX had no polymerase activity (Figure 4E, lanes 1–3). Previously, a short-patch (one nucleotide) DNA extension by *D. radiodurans* PolX was detected in the presence of a large excess of unlabeled DNA substrate that was added together with dNTPs (to prevent multiple rounds of enzyme dissociation/association) (31). However, we could not detect any DNA polymerase activity in these conditions with our PolX samples (Supplementary Figure S4F). This suggested that the previously observed activity might have resulted from an admixture of other DNA polymerase(s) in the PolX preparations (31).

In the presence of Mn^2+^, both noncanonical deinococcal PolXs revealed robust 3′-5′ exonuclease activity on all types of substrates, resulting in rapid shortening of the 5′-labeled primer (Figure 4C, lanes 13–24; Figure 4E, lanes 4–6). In comparison, only low level of exonuclease activity was observed in the presence of Mg^2+^ (lanes 11–12 in Figure 4C,E and Supplementary Figure S4A, B). Titration experiments demonstrated that the optimal concentration of Mn^2+^ for this activity was between 3 and 10 mM, while no efficient cleavage was observed at any tested Mg^2+^ concentration (Figure 4I). In agreement with our observations, previously investigated bacterial PolXs, including PolX from *D. radiodurans*, were shown to possess Mn^2+^-dependent exonuclease activities (23,25,33,41,83). To confirm that this activity depends on the PHP domain, we obtained a mutant variant of *D. radiodurans* PolX with alanine substitutions of two of the nine active site residues in PHP involved in Mn^2+^ binding (H332A/H334A). As expected, the mutant PolX lacked the exonuclease activity in the presence of either Mg^2+^ or Mn^2+^ (Figure 4F). At the same time, alanine substitutions in the polymerase active site did not affect the exonuclease activity (Figure 4D, lanes 13–24).

For comparison, we tested the activities of *B. subtilis* PolX in the same conditions. It was found that it can efficiently extend DNA with both Mg^2+^ and Mn^2+^. In the presence of Mg^2+^, the major reaction product at low PolX concentration corresponded to the addition of a single nucleotide to the primer 3′-end (Figure 4G, lanes 1-12), while it was further extended at high PolX concentration (Supplementary Figure S4C, lanes 1–12). In the presence of Mn^2+^, *B. subtilis* PolX revealed highly efficient 3′-5′ exonuclease activity, which competed with primer extension (Figure 4G, lanes and Supplementary Figure S4C, lanes 13–24). The mutant *B. subtilis* PolX with substitution in the polymerase active site (D203A) completely lost its polymerase activity but remained active as exonuclease (Figure 4H). This confirms that the PHP domain, but not the polymerase active site, is responsible for the 3′-5′ exonuclease activity in both canonical and noncanonical PolX polymerases.

### Association of prokaryotic PolX genes with components of DNA repair pathways

Eukaryotic PolXs, including Polλ and Polμ, participate in the NHEJ pathway by performing limited DNA synthesis at the gaps during DSB repair (84). Many bacteria encode components of the NHEJ pathway, including the Ku protein, homologous to eukaryotic Ku, and Ligase D (LigD), a multifunctional factor with ligase, polymerase and sometimes nuclease (phosphoesterase) activities (85). To uncover whether prokaryotic PolXs might be connected to DSB repair, we analysed the presence of PolX, Ku and LigD in fully sequenced bacterial genomes (24973 genomes in total). To avoid biases in the frequencies of PolXs and NHEJ components in bacterial genomes due to highly uneven numbers of sequenced genomes in various lineages (see the first section of Results), we generated a non-redundant sample of 2826 representative genomes containing 452 PolX variants (332 canonical and 114 altered), using a previously described algorithm of genome clustering (52). This algorithm allows to obtain a representative collection of genomes based on their sequence diversity and not on taxonomy, thus helping to smooth possible taxonomic biases.

**Figure 5.**
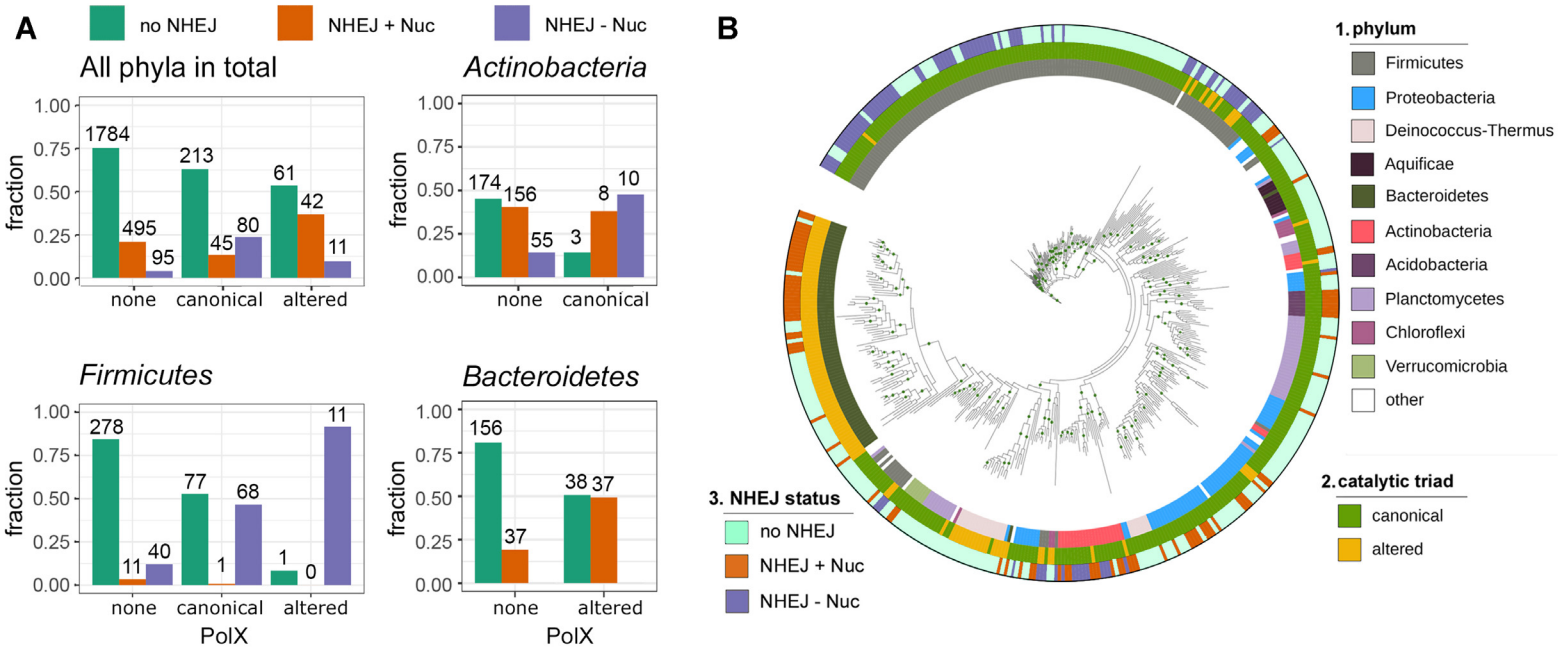
Co-occurrence of PolXs with components of the NHEJ pathway in bacterial genomes. (**A**) Distribution of the NHEJ genes in the non-redundant sample of 2826 complete bacterial genomes either lacking PolX, or containing canonical or altered PolXs, shown for all bacterial phyla or individually for *Firmicutes, Actinobacteria* and *Bacteroidetes*. For *Actinobacteria*, only canonical PolXs are shown due to the very small number of genomes with altered PolX in this phylum (3 genomes in the non-redundant sample used for analysis). For *Bacteroidetes*, only altered PolXs are shown due to the absence of genomes with canonical PolX in the non-redundant sample. The proportion of genome variants with each gene combination is shown on the ordinate axis. The numbers of genomes in each group are indicated. (**B**) Co-occurrence of PolXs with NHEJ genes in the non-redundant set of bacterial genomes, shown on a phylogenetic tree generated for the 452 PolX sequences found in these genomes (note that the tree topology is different from Figure 2A due to the much smaller number of PolX sequences used for analysis; this tree is used to illustrate solely the diversity of combinations of PolX and NHEJ in different phyla, and not the evolution of PolXs). The rings are annotated as follows: 1, phylum (the color code corresponds to Figure 2A); 2, catalytic triad status (green, canonical; ochre, altered); 3, NHEJ status (ligh green, no NHEJ; orange, NHEJ+Nuc; violet, NHEJ-Nuc). The green dots on the nodes correspond to bootstrap values of 98–100.

We then analyzed co-occurrences of PolX and NHEJ genes in the non-redundant sample of genomes (Figure 5). For comparison, a similar analysis was also performed for the complete set of sequenced genomes (Supplementary Figure S5A). We defined functional LigD variants as those containing both ligase and polymerase domains in the same protein, and looked separately for LigD variants with or without the nuclease domain. The genomes that contained both Ku and any of these two LigD variants were classified as encoding the NHEJ pathway with and without associated nuclease activities, respectively (NHEJ+Nuc and NHEJ-Nuc). The genomes that contained either Ku or LigD alone, or lacked both proteins were classified as lacking the NHEJ pathway.

It was found that 72.8% of the non-redundant genomes (2058 out of 2826) lack the canonical NHEJ pathway; most of them lack both Ku and LigD and some contain only Ku or LigD domains alone. Among them, canonical and altered PolXs are present in 10.3% and 3.0%, respectively. The absolute numbers of genomes in each group are indicated in Figure 5A (all phyla). Furthermore, 20.6% of the genomes (582 out of 2826) belong to the NHEJ+Nuc group and 6.6% (186 out of 2826) belong to the NHEJ-Nuc group. In the NHEJ+Nuc group, canonical and altered PolXs are present in 7.7% and 7.2% of the genomes, which is comparable to the NHEJ-minus genomes (Figure 5A). In contrast, in the NHEJ-Nuc group they are present in 43% and 5.9% of the genomes, respectively. Thus, the NHEJ-Nuc genomes are enriched with both canonical and noncanonical PolX variants, and in sum about half of the NHEJ-Nuc genomes contain PolXs (in comparison with 13.3% of NHEJ-minus genomes) (Figure 5A). Analysis of the complete set of sequenced genomes gives similar results, although with slightly different proportions of genomes in each group (Supplementary Figure S5A). This indicates that the nuclease activity of PolXs might compensate for the absence of the nuclease domain in LigD encoded in the NHEJ-Nuc genomes.

From the other side, PolX-containing genomes are generally enriched with NHEJ genes. Either NHEJ+Nuc or NHEJ-Nuc pathways are found in 37% of genomes encoding canonical PolXs and 46.5% of genomes encoding altered PolXs, in comparison with 24.9% of PolX-minus genomes (Figure 5). Importantly, genomes containing altered PolXs more often encode NHEJ components than genomes with canonical PolXs (46.5% versus 37%) (Figure 5A). This difference is even higher when considering all sequenced genomes (54.8% versus 30.4%) (Supplementary Figure S5A). Both NHEJ pathways are enriched in the genomes containing altered PolX variants (36.8% and 9.6% for NHEJ+Nuc and NHEJ-Nuc, respectively, versus 20.9% and 4.0% in the PolX-minus genomes) (Figure 5A).

The distribution of the NHEJ pathways and PolX variants is uneven between bacterial phyla (Figure 5A, B, Supplementary Figure S5A). We therefore performed analysis of PolX frequencies separately in three bacterial phyla with many sequenced genomes encoding PolXs, *Actinobacteria*, *Firmicutes* and *Bacteroidetes* (Figure 2A). *Firmicutes* encode almost exclusively the NHEJ-Nuc pathway (found in 24% of non-redundant genomes) and canonical PolX variants (30% of genomes). The NHEJ-Nuc pathway is strongly enriched in genomes containing canonical PolXs (46.6% in comparison with 12.2% of genomes lacking PolXs) (Figure 5A). While the inactive PolX variants are much less common, they are almost always associated with the NHEJ-Nuc pathway (11 out of 12 genomes encoding altered PolXs) (Figure 5A). *Actinobacteria* often encode either the NHEJ+Nuc pathway (41% of sequenced genomes) or the NHEJ-Nuc pathway (16% of genomes). Only a small fraction of this phylum encodes PolXs (6% of genomes) and almost all identified PolX variants are canonical (Figure 5A,B). The proportion of genomes encoding the NHEJ+Nuc pathway is similar among the genomes lacking or containing PolXs. However, the NHEJ-Nuc pathway is found much more frequently in the genomes encoding PolXs, suggesting a functional association (Figure 5A). *Bacteroidetes* usually encode the NHEJ+Nuc pathway (found in 28% of sequenced genomes) and noncanonical PolX variants (also in 28% of genomes). In this phylum, PolXs are also strongly associated with the NHEJ pathway (∼49% of PolX-containing genomes encode NHEJ versus ∼19% of genomes lacking PolXs) (Figure 5A). Thus, it can be concluded that PolX genes are often co-encoded with the NHEJ genes in different bacterial phyla but the type of association between different PolX and NHEJ groups is specific for individual phyla.

To estimate statistical significance of the found associations, we compared the observed frequencies of PolX and NHEJ genes with their expected distributions for random association and calculated corresponding *P*-values using the Pearson χ-square test of independence. It was found that the genomic association of PolXs with NHEJ is highly statistically significant (*P*-value = 2.3e^–10^ for co-occurrence of both PolX variants with both NHEJ pathways). Highly significant non-random associations were also observed when considering the two types of PolXs independently, either for all genomes or for individual bacterial phyla (Supplementary Figure S6).

To test whether the observed enrichment of the NHEJ pathways in the PolX-encoding genomes might be simply explained by a larger size of these genomes, we compared genome lengths depending on the presence of NHEJ and PolX for the same sample of non-redundant genomes. It was found that NHEJ-containing genomes are indeed on average larger than genomes without NHEJ (Supplementary Figure S5B) (86). However, a similar trend was observed for genomes both lacking and encoding PolXs. Moreover, in the case of genomes containing the NHEJ-Nuc pathway the length of the genomes with PolXs was even somewhat smaller than in the case of genomes lacking PolXs (Supplementary Figure S5B). It is therefore unlikely that the genomic association of PolXs and NHEJ pathways can be a nonspecific event resulting from increased lengths of such genomes.

To better understand possible biological functions of prokaryotic PolXs, we also analyzed operon structures of canonical and altered PolXs and identified most common Pfam domains enriched in proteins encoded in the genomic neighborhood of the PolX genes (see Materials and Methods). It was found that the gene neighborhood of polymerases is specific for each investigated class of organisms (Supplementary Figure S7 and Supplementary Table S3). However, some of the detected genetic associations may suggest a possible functional connection between PolX and nucleic acid processing. Remarkably, in the class *Alphaproteobacteria* the most abundant operon neighbor of noncanonical PolXs is LigD, confirming a functional connection between PolX and NHEJ (Supplementary Figure S7 and Supplementary Table S3) (87–89). In the classes *Bacilli* and *Clostridia*, the most frequent operon neighbors of both canonical and non-canonical PolX include proteins ZapA, which participates in the Z-ring formation and synchronizes cell division with chromosome segregation (90–93), and a MutS2 nuclease, which may be involved in processing of recombination intermediates and natural transformation in *B. subtilis* (94,95). The PolX operons in *Bacilli* also often contain a pore forming protein Colicin V, suggesting that together these proteins may promote gene exchange between bacteria. Furthermore, a common gene neighbor of altered PolXs in *Deinococci* is a stand-alone PHP domain, which might provide additional nuclease activities during DNA repair. Finally, canonical PolXs in Archaea are strongly associated with an ATP-dependent DNA ligase in the class *Methanomicrobia* and with a Mut7-C domain-containing RNase in the class *Halobacteria* (96) (Supplementary Table S3). Overall, this analysis suggests that no universal genetic associations are characteristic for PolX operons but some of them may have dedicated functions in nuclear acid processing and genomic DNA repair.

Intriguingly, we also found a substantial number of genomes that contained more than one PolX gene (121 genomes among the 6239 genomes containing 6362 unique PolX sequences and 87 genomes among the 2846 genomes containing 2935 non-redundant PolX variants) (Supplementary Table S4). Most of them contained 2 PolX genes and two genomes of *Bacteroidetes* contained three PolX genes. Half of them contained canonical and noncanonical PolX genes at the same time (Supplementary Table S4). These variants likely correspond to independently acquired genes *via* horizontal gene transfer since the majority of such polymerase pairs are located in very distant clades of the PolX tree. This suggests that canonical and altered polymerases might play different functions in these bacterial species.

## DISCUSSION

Our analysis of prokaryotic PolXs showed that they are much more diverse than their eukaryotic counterparts and form several clades including canonical Polβ-like polymerases and highly divergent noncanonical PolX polymerases. Characteristic features of noncanonical PolX include: (i) substitutions of the catalytic triad residues in the polymerase active site in the palm domain, (ii) deletions in the palm domain; (iii) substitutions of conserved residues of the dNTP binding site at the interface of the palm and fingers domains and (iv) deletions in the fingers domain (Figure 1B). Since the aspartate triad is essential for the catalytic activity in Polβ and its relatives (35,36,42,79,80), noncanonical PolXs probably lack a DNA polymerase activity and are unlikely to act as DNA polymerases. Furthermore, alterations in the palm and fingers domains in these PolXs often accompany each other confirming that the mutated elements are no longer important for the polymerase activity. Indeed, our analysis of two noncanonical PolXs from *D. radiodurans* and *D. gobiensis* demonstrated that they are inactive as DNA polymerases.

Despite dramatic changes in the structure of the polymerase active site, the noncanonical PolXs do not have specific alterations in the N-terminal dRP-lyase and C-terminal PHP exonuclease domains. In particular, the majority of prokaryotic PolXs contain a highly conserved PHP domain with the predicted exonuclease activity. Indeed, we demonstrated that noncanonical PolXs from *D. radiodurans* and *D. gobiensis* have a high level of Mn^2+^-dependent 3′-5′ exonuclease activity, which is abrogated in the presence of mutations of conserved residues involved in the catalytic metal binding in the PHP domain. Since the PHP domain has a broad range of activities toward 3′-ends and AP-sites in DNA substrates of various structures (23,33,97), both canonical and altered PolXs might participate in the sanitization of the primer 3′-ends during DNA replication and break repair, and possibly in the processing of DNA intermediates during BER.

In the complex of *T. thermophilus* PolX with DNA, the PHP domain is remote from the DNA substrate and the mode of its interactions with DNA during exonucleolytic reaction remains unknown (Figure 1D). Interestingly, available structure of the noncanonical PolX from *D. radiodurans* demonstrates significant conformational changes in comparison with the *T. thermophilus* PolX (Figure 1E). It can be speculated that such changes may be important for switching the activities of PolXs, but their functional role remains to be established. The coordination of various activities in canonical PolXs, as well as the role of polymerase domains in the nuclease activity of noncanonical PolXs, will be important questions for further studies.

Most noncanonical PolXs, including PolXs found in the *Bacteroidetes* and *Deinococcus-Thermus* phyla, form a single group on the phylogenetic tree suggesting their common evolutionary origin (Figure 2A). While more sophisticated analysis is needed to understand the exact evolutionary origins of altered PolXs, the presence of related PolXs in unrelated bacteria phyla indicates their horizontal transfer between prokaryotic species. It should be noted that noncanonical PolXs can also be present together with canonical PolXs in some genomes, suggesting that cooperation between the two types of DNA polymerases may be beneficial for host species.

Bacterial genomes encoding both canonical and noncanonical PolXs are enriched with genes encoding the main components of bacterial NHEJ, the Ku protein and the multifunctional ligase LigD (87,88). Interestingly, genomes with altered PolXs encode NHEJ pathways even more frequently than genomes with canonical PolXs, suggesting that the polymerase activity of PolX may not be important for NHEJ. Furthermore, both canonical and altered PolXs can be associated with the NHEJ-Nuc pathway, in which LigD lacks the nuclease domain involved in processing of DNA ends. In this case, the exonuclease activity of PolX might compensate for the absence of the nuclease activity in LigD during NHEJ. At the same time, altered PolXs are also frequently found in the same genomes with the NHEJ+Nuc pathway indicating that they might still have a role in DNA repair even in the presence of the nuclease activity in LigD.

Analysis of the genomic neighborhood of prokaryotic PolXs also reveals their association with nucleic acid processing enzymes in some bacterial classes, including LigD in *Alphaproteobacteria*, suggesting their possible functional cooperation. Therefore, PolX family polymerases may be generally involved in double-strand break repair, in particular NHEJ, in both bacteria and eukaryotes (27), suggesting that this PolX function may have first appeared in the prokaryotic world. Intriguingly, however, the polymerase activity of PolX is apparently not important for NHEJ in bacteria, which may be compensated by the polymerase domain of LigD. In contrast, PolXs involved in eukaryotic NHEJ lack the exonuclease domain, which is obligatorily present in prokaryotic PolXs, and cooperate with additional exonucleases (98).

Recent analysis revealed another example of inactive DNA polymerase from the Y family, ImuB, which forms a multisubunit complex with a homolog of the Pol III alpha subunit DnaE2 and a RecA homolog ImuA and interacts with the processivity clamp (99–101). This complex was proposed to act as a mutasome due to the error-prone catalytic activity of DnaE2 while ImuB serves as an organizing subunit. In comparison, inactive PolX polymerases may both play architectural and DNA binding functions during nonhomologous end joining and also directly contribute to DNA processing.

Available data from *in vivo* experiments, while very limited, suggest that PolXs may have different functions in different bacteria. Thus, PolXs from *B. subtilis* and *T. thermophilus* were proposed to participate in BER, while PolX from *D. radiodurans* was shown to be important for radioresistance and genome recovery after γ-irradiation, but not take part in the BER or nucleotide excision repair pathways (26,33,34,102,103). In *D. radiodurans*, PolX and the SbcCD nuclease, an evolutionary conserved structure-specific nuclease involved in processing of double-strand breaks, were shown to play complementary roles during post-radiation repair, suggesting their involvement in the same repair pathway (103). While the biological functions of most prokaryotic PolXs remain to be established, we hypothesize that the main role of both canonical and noncanonical polymerases may be in the processing of DNA intermediates during DNA repair rather than in DNA synthesis. Investigation of their cellular roles, including proposed participation in the NHEJ pathway, and of their interplay with other DNA repair pathways will be an important goal of future research.

## DATA AVAILABILITY

All primary data are available from the corresponding authors upon request.

## Supplementary Material

gkac461_Supplemental_Files
