# Supplementary material for: Noncanonical prokaryotic X family DNA polymerases lack polymerase activity and act as exonucleases
Source: Nucleic Acids Res. 2022 Jun 3;50(11):6398–413. doi: 10.1093/nar/gkac461 (PMC9226535; doi:10.1093/nar/gkac461)
Supplement: gkac461_Supplemental_Files [file gkac461_supplemental_files.zip › Prostova_SI.pdf]

Noncanonical prokaryotic X family DNA polymerases lack polymerase activity and act as exonucleases

Maria Prostova, Evgeniy Shilkin, Alexandra Kulikova,

Alena Makarova, Sergei Ryazansky, Andrey Kulbachinskiy

SUPPLEMENTARY INFORMATION

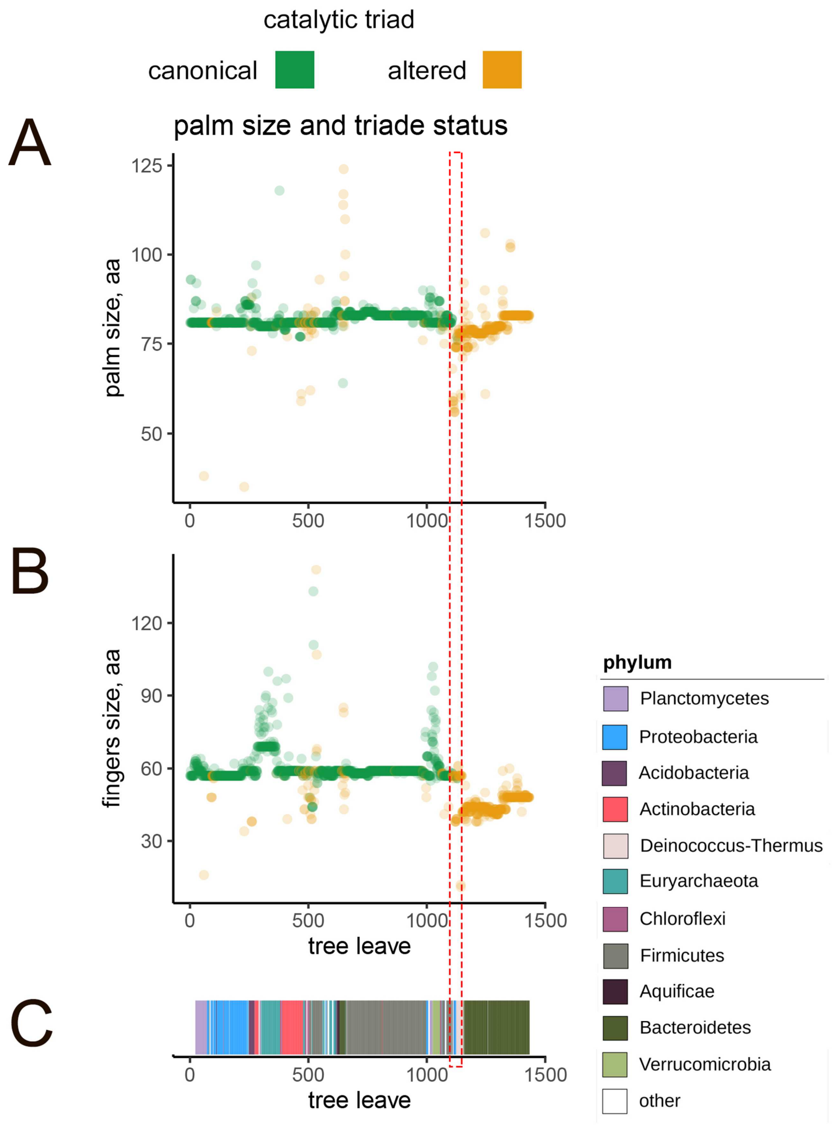

Figure S1. Distribution of the lengths of the palm and fingers domains in canonical and altered PolXs. (A) Palm domain length of PolXs plotted for the phylogenetic tree shown in Figure 2A. The length of the palm domain was calculated as the number of residues between conserved motifs corresponding to 181-AGSLRR-186 and 256-FTGSKDHN-263 of *B. subtilis* PolX (including the motif sequences). The group of PolXs with the shortest palm domains (<65 residues) is shown with a red box. (B) Fingers domain length. The length of the fingers domain was calculated as the number of residues between conserved motifs corresponding to 256-FTGSKDHN-263 (the end of palm) and 307-LIPPEIRE-314 of *B. subtilis* PolX. Three PolXs have fingers size of <10 residues (from *Deinococcus proteolyticus*, *Deinococcus radiophilus* and unclassified bacterium D16-56); these proteins also have extra small palm sizes with altered catalytic triads, while preserving the full-length PHP domain. The large size of fingers (>100 residues) in some PolXs is due to an elongated linker between the fingers and PHP domains, which is included in calculation. (C) Distribution of prokaryotic phyla with the tree leave number, the color code corresponds to Figure 2A.

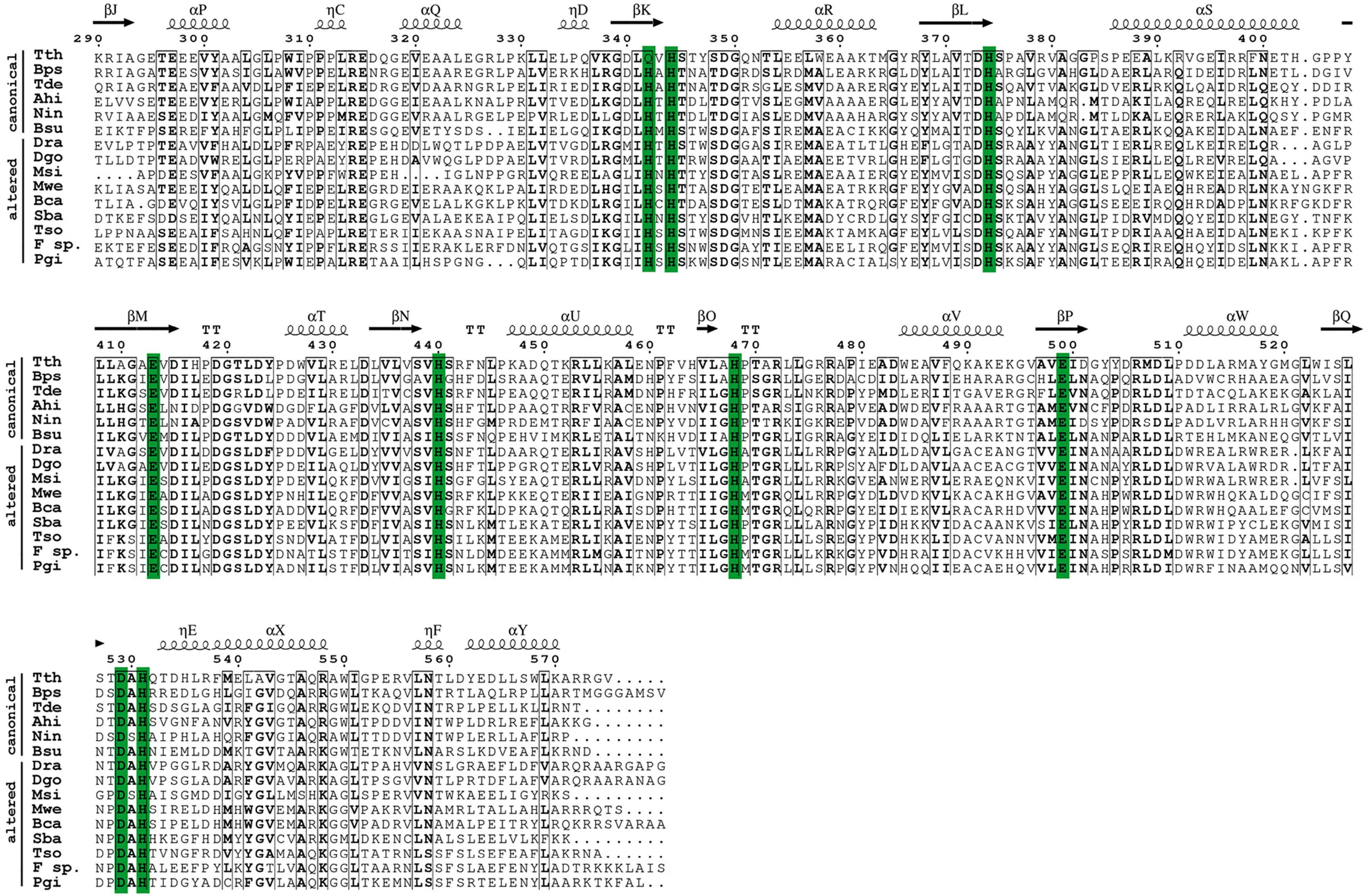

Figure S2. Alignment of the sequences of the PHP domain in prokaryotic PolXs. The active site residues are shown in green. Similar residues (similarity score > 0.7) are shown in bold. Amino acid numbering and the secondary structure of the PHP domain is shown above the alignment for *T. thermophilus* PolX (based on the structure from PDB: 3AU0). The abbreviations of the species names are the same as in Figure 3A.

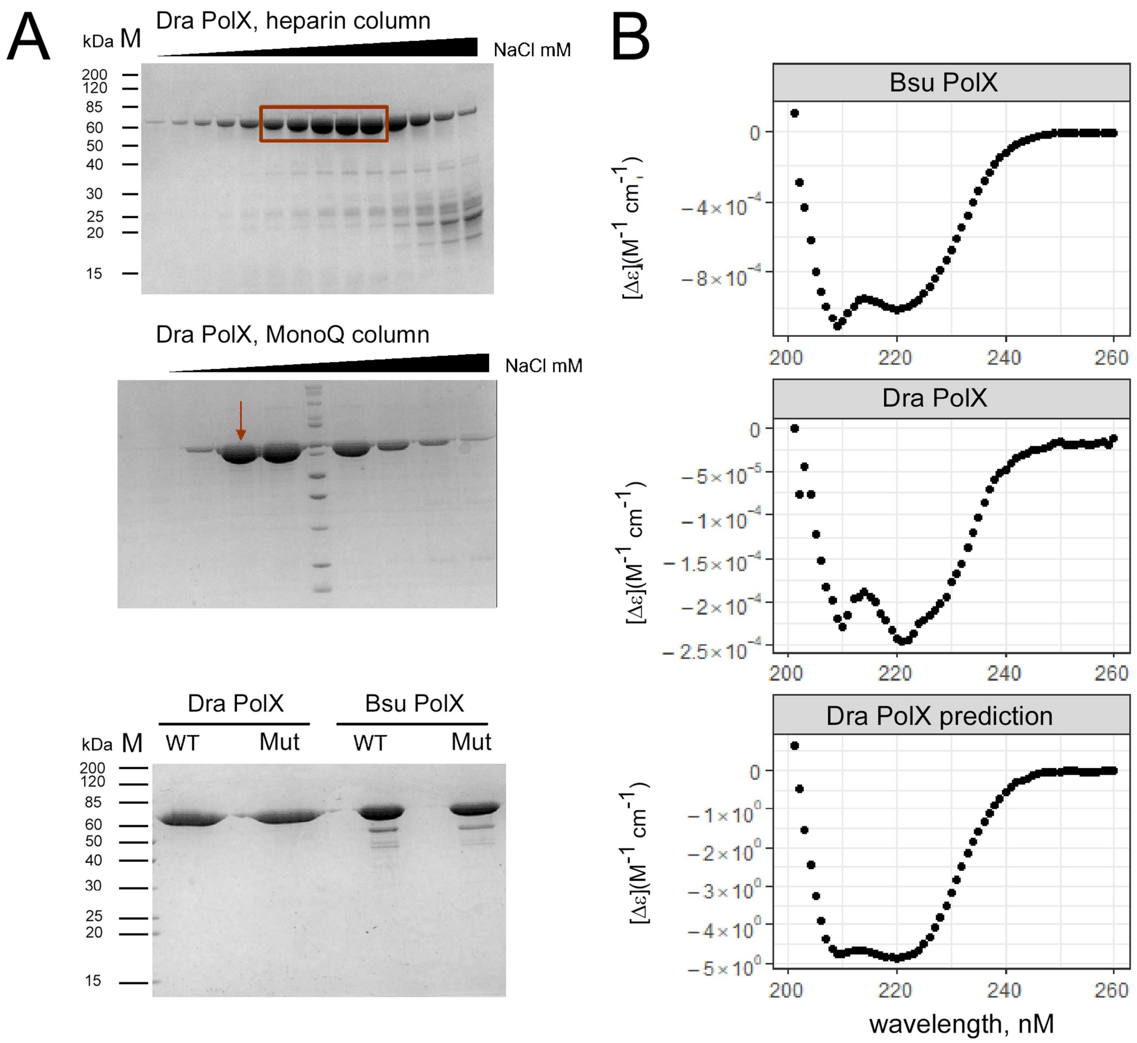

Figure S3. Purification of PolX polymerases. (A) Analysis of chromatographic fractions during purification of *D. radiodurans* PolX on a heparin column (top, collected fractions are shown with a red box) and a MonoQ column (middle, collected fraction is shown with an arrow). The final purified samples of *D. radiodurans* and *B. subtilis* PolX are shown on the bottom gel. The lengths of molecular size markers are indicated on the left. (B) Circular dichroism spectra for PolXs from *B. subtilis* (top) and *D. radiodurans* (middle). The predicted spectrum for *D. radiodurans* PolX based on the content of α and β structures in its published structure (PDB: 2W9M) is shown on the bottom.

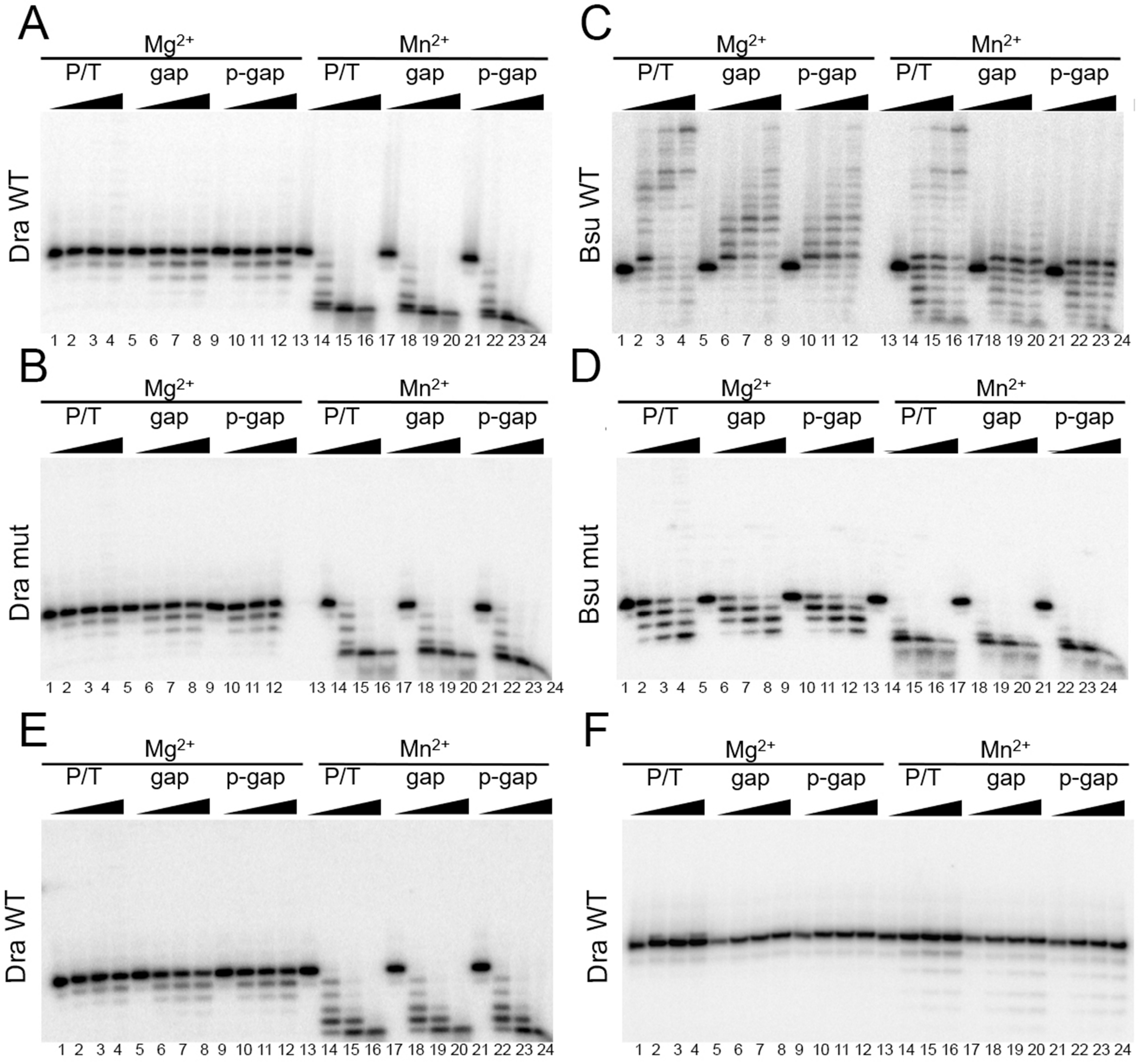

Figure S4. Catalytic activity of *D. radiodurans* and *B. subtilis* PolXs under different conditions. (A,B) Analysis of the activities of wild-type *D. radiodurans* PolX (A) and its mutant variant with substitutions in the polymerase active site (B) measured at high polymerase concentration (1 μM) in the presence of 200 μM dNTP substrates. The reactions were performed at 30°C for 0, 10, 30, 90 min. (C,D) The same for *B. subtilis* PolX and its catalytic mutant. (E) Analysis of the activity of wild-type *D. radiodurans* PolX (20 nM) at low dNTP concentration (10 μM). (F) Analysis of the activity of wild-type *D. radiodurans* PolX (1 μM) in the presence of unlabeled competing DNA substrate, using the conditions from ref. 31 (see Materials and Methods for details). Experiments in panels A-D were independently repeated two times, experiments in panels E and F were performed without replicates.

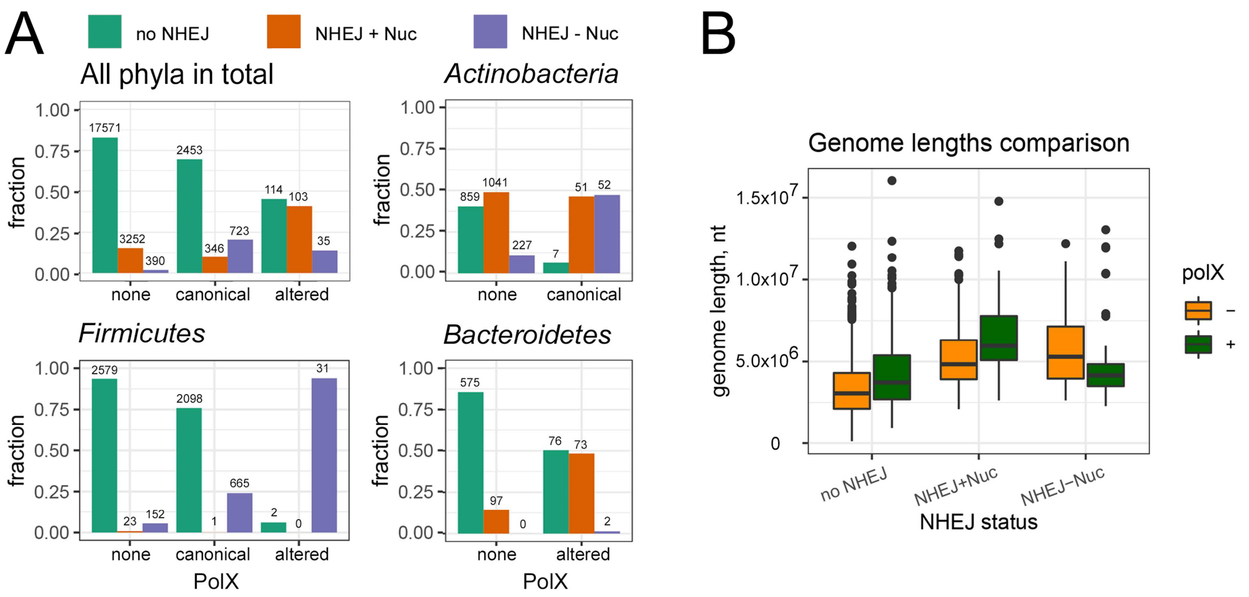

Figure S5. (A) Co-occurrence of PolXs with components of the NHEJ pathway in fully-sequenced bacterial genomes. The plots show distribution of the NHEJ genes in all fully sequenced bacterial genomes (24973 in total) either lacking PolX, or containing canonical or altered PolXs, shown for all bacterial phyla or individually for *Firmicutes, Actinobacteria* and *Bacteroidetes*. For *Actinobacteria*, only canonical PolXs are shown due to the very small number of genomes with altered PolX in this phylum (4 genomes in the complete set of genomes used for analysis). For *Bacteroidetes*, only altered PolXs are shown due to the very small number of genomes with canonical PolX (4 genomes). The proportion of genome variants with each gene combination is shown on the ordinate axis. The total numbers of genomes in each group are indicated. (B) Genome size distribution for genomes without complete NHEJ, with NHEJ+Nuc, or with NHEJ-Nuc, depending on the presence of PolX, shown for the non-redundant sample of 2826 representative genomes.

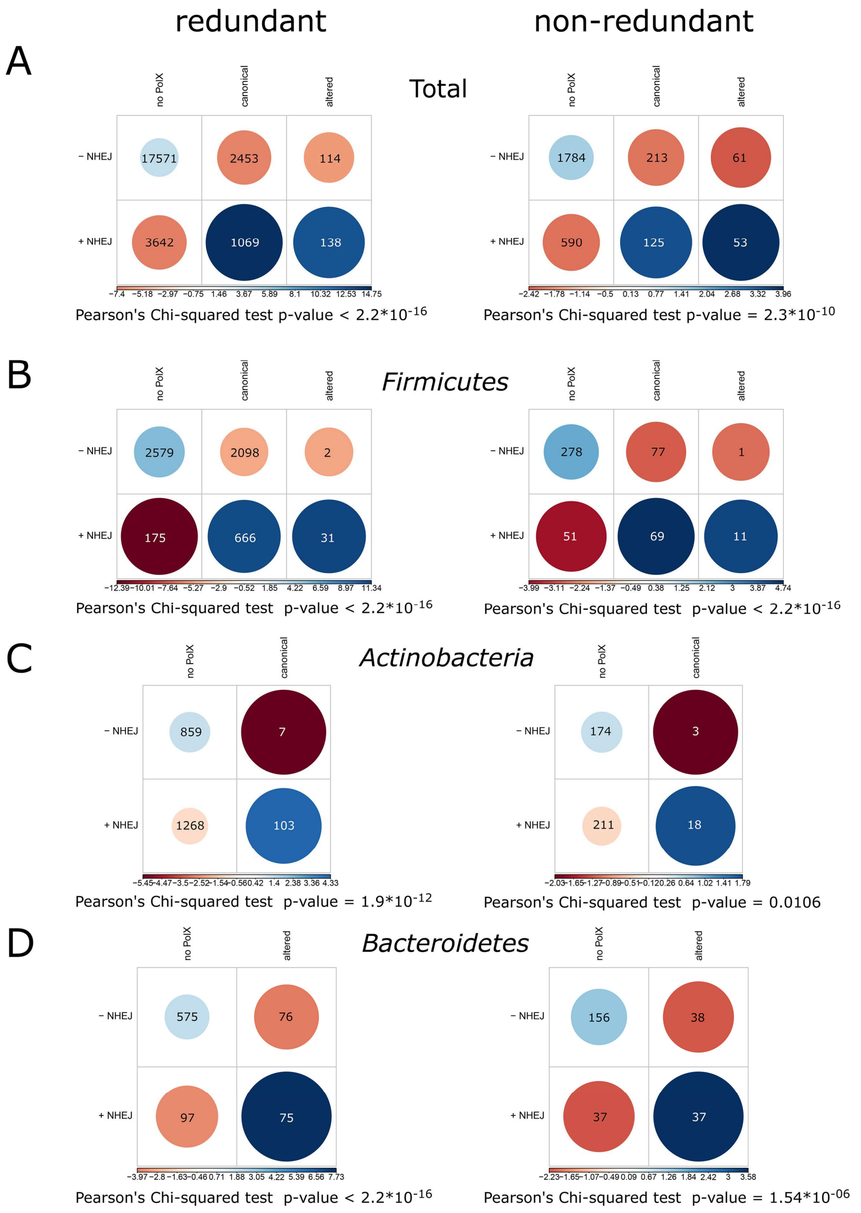

Figure S6. Correlation plots illustrating co-occurrence of canonical or altered PolX with NHEJ proteins (Ku and LigD with or without the nuclease domain) in bacterial genomes from different phyla. A – all phyla together, B – genomes of the *Firmicutes* phylum, C – genomes of *Actinobacteria*, D – genomes of *Bacteroidetes*. For *Actinobacteria*, only canonical PolXs are shown due to the very small number of genomes with altered PolX in this phylum. For *Bacteroidetes*, only altered PolXs are shown due to the very small number of genomes with canonical PolX. Correlation plots were built using the Chi-square test of independence and corrplot package implemented in R using corresponding contingency tables for redundant (*left*) and non-redundant (*right*) collections of full bacterial genomes. The numbers of genomes of each type are shown inside the circles. The size and color of each circle show its relative contribution to the total significance score. Blue and red circles represent positive and negative association, respectively, between the row and column variables. The color bar under each plot shows the scale of standardized Pearson residuals. The p-value from the Chi-square test of independence is indicated under each plot. Larger and darker circles correspond to genome types that are more strongly over-represented (blue) or under-represented (red) in comparison with expected numbers for random distribution.

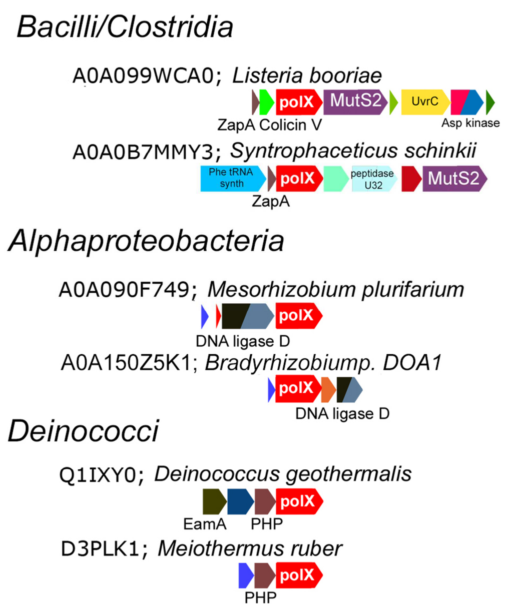

Figure S7. Operon structure of representative canonical PolXs from *Bacilli/Clostridia* and noncanonical PolXs from *Alphaproteobacteria* and *Deinococci*. The PolX gene is in red, the most common genome neighbors are indicated.

Table S1. Most abundant triad variants in prokaryotic PolXs.

Triad	canonical			
	DDD	DDE	DEE	EDE
# of seqs	2102	21	2	1
%	98.87	0.99	0.09	0.05

Triad	altered												
	EEK	TQE	DAQ	EVD	TRE	DAR	EER	VRE	NDR	DTQ	EEI	TTE	EEV
# of seqs	51	40	32	23	22	21	21	14	13	12	12	12	11
%	6.30	4.94	3.96	2.84	2.72	2.60	2.60	1.73	1.61	1.48	1.48	1.48	1.36

Table S2. Conservation of the key residues in the dNTP-binding pocket of canonical and altered prokaryotic PolX polymerases.

Pol β	Function	Canonical	Altered	*B. subtilis*	*T. thermophilus*	*D. radiodurans*
S180 (palm)	Coordinates Pγ	S 99.5%	D 33%, E 26%, A 14%, Q 12%	S183	S188	D187
R183 (palm)	Coordinates Pβ, stabilizes 274-GS-275	R 100%	R 97.2%	R186	R191	R190
G189 (palm)	Stabilizes dNTP and the primer 3’-end in *B. subtilis* PolX	K 36%, G 58%	D 25%, E 23%, G 12%, S 12%	K192	G197	R196
271-YF-272 (fingers)	Steric gate, D192 coordination	YF 54.3%, HF 40%	no conservation	255-HF-256	258-YL-259	249-LR-250
274-GS-275 (fingers)	Cis-peptide bond	97.6% GS	GS 42%, GN 12%, AA 7.9%, AS 7.3%	258-GS-259	261-GS-262	251-SG-252
D276 (fingers)	dNTP interaction	K 84.8 %, R 6.8%, Q 5.3%	E 24%, A 21.5%, P 15%	K260	K263	E253
N279 (fingers)	dNTP interaction	N 92.87%	L 47.7%, V 20.5%, I 14%	N263	S266	A256

Table S3. Abundant genome neighbors of the PolX genes of different bacterial classes.

Class	PolX triad status	Protein family	# of genomes	Total # of genomes in the class	Fraction
Acidithiobacillia	canonical	Phosphoribosyl pyrophosphate synthase	4	4	1.00
Alphaproteobacteria	altered	DNA ligase D (ATP)	9	10	0.90
Bacilli	canonical	Thioredoxin	712	1372	0.52
		Cell division protein ZapA	1321		0.96
		UvrABC system protein UvrC	349		0.25
		Transmembrane DUF350	477		0.35
		Colicin V production family protein	1349		0.98
		Endonuclease MutS2	1338		0.98
Betaproteobacteria	canonical	Outer membrane efflux family protein	124	190	0.65
		Multicopper oxidase family protein	106		0.56
		RNA polymerase-binding transcription factor DksA	61		0.32
Chitinophagia	altered	50S ribosomal protein L27	8	9	0.89
Clostridia	canonical	Endonuclease MutS2	9	23	0.39
		Phenylalanine--tRNA ligase beta subunit	7		0.30
		Phenylalanine--tRNA ligase alpha subunit	7		0.30
		Cell division protein ZapA	13		0.57
Clostridia	altered	Cell division protein ZapA	5		0.22
		Phenylalanine--tRNA ligase beta subunit	8		0.35
		Phenylalanine--tRNA ligase alpha subunit	7		0.30
Cytophagia	altered	ADP-heptose:LPS heptosyl transferase	26	26	1.00
Deinococci	altered	PHP domain-containing protein	10	12	0.83
Deltaproteobacteria	canonical	CAAX amino terminal protease	12	13	0.92
Halobacteria	canonical	Mut7-C domain-containing protein	49	49	1.00
Methanomicrobia	canonical	DNA ligase (ATP)	22	22	1.00
Sphingobacteriia	altered	ADP-heptose:LPS heptosyl transferase	13	17	0.76

		Thioredoxin reductase	4		0.24
		Peptidase_S49 domain-containing protein	9		0.53
		Histidine phosphatase super family protein	8		0.47

Table S4. Number of genomes in the RefSeq database that contain more than one PolX gene.

**Phylum**	**all noncanonical**	**canonical and noncanonical**	**all canonical**	**Total**
Acidobacteria	0	2	1	3
Actinobacteria	0	5	7	12
Bacteroidetes	6	17	2	25
Balneolaeota	0	0	1	1
Caldiserica	0	1	0	1
Chloroflexi	0	2	0	2
Deinococcus-Thermus	0	5	0	5
Euryarchaeota	0	8	4	12
Firmicutes	1	1	3	5
Planctomycetes	0	0	1	1
Proteobacteria	13	3	4	20
**Total**	20	44	23	87
